# Ligation‐based assay for variant typing without sequencing: Application to SARS‐CoV‐2 variants of concern

**DOI:** 10.1111/irv.13083

**Published:** 2022-12-12

**Authors:** Dalton J. Nelson, Meghan H. Shilts, Suman B. Pakala, Suman R. Das, Jonathan E. Schmitz, Frederick R. Haselton

**Affiliations:** ^1^ Department of Biomedical Engineering Vanderbilt University Nashville Tennessee USA; ^2^ Department of Medicine Vanderbilt University Medical Center Nashville Tennessee USA; ^3^ Department of Pathology, Microbiology and Immunology Vanderbilt University Medical Center Nashville Tennessee USA; ^4^ Vanderbilt Institute for Infection, Immunology and Inflammation Vanderbilt University Medical Center Nashville Tennessee USA

**Keywords:** genetics, ligation, PCR, sequence‐free, single‐nucleotide polymorphisms (SNPs), variants

## Abstract

**Background:**

COVID‐19 prevalence has remained high throughout the pandemic with intermittent surges, due largely to the emergence of genetic variants, demonstrating the need for more accessible sequencing technologies for strain typing.

**Methods:**

A ligation‐based typing assay was developed to detect known variants of severe acute respiratory syndrome virus 2 (SARS‐CoV‐2) by identifying the presence of characteristic single‐nucleotide polymorphisms (SNPs). General principles for extending the strategy to new variants and alternate diseases with SNPs of interest are described. Of note, this strategy leverages commercially available reagents for assay preparation, as well as standard real‐time polymerase chain reaction (PCR) instrumentation for assay performance.

**Results:**

The assay demonstrated a combined sensitivity and specificity of 96.6% and 99.5%, respectively, for the classification of 88 clinical samples of the Alpha, Delta, and Omicron variants relative to the gold standard of viral genome sequencing. It achieved an average limit of detection of 7.4 × 10^4^ genome copies/mL in contrived nasopharyngeal samples. The ligation‐based strategy performed robustly in the presence of additional polymorphisms in the targeted regions of interest as shown by the sequence alignment of clinical samples.

**Conclusions:**

The assay demonstrates the potential for robust variant typing with performance comparable with next‐generation sequencing without the need for the time delays and resources required for sequencing. The reduced resource dependency and generalizability could expand access to variant classification information for pandemic surveillance.

## 1 INTRODUCTION

As the COVID‐19 pandemic has progressed, genetic variants of the severe acute respiratory syndrome virus 2 (SARS‐CoV‐2) have emerged presenting new challenges in combatting the virus and effectively prolonging the pandemic.^1^ In particular, some variants of concern (VOCs) demonstrate increased disease severity, have greater transmissibility, and interfere with vaccines, treatments, and diagnostic tests.^1^, ^2^, ^3^, ^4^, ^5^, ^6^ Real‐time monitoring and tracking of the genetic progression are essential for informing dynamic adaptation to the changing needs required to counter the pandemic. The gold standard for the discovery of emerging variants and surveillance of existing variants is viral genome sequencing.^7^, ^8^, ^9^, ^10^ Although sequencing is necessary for discovering new variants, as a method to classify variants, the strategy is expensive and requires access to high‐level technical expertise and sequencing equipment.

To address the greater need and demand for population‐level SARS‐CoV‐2 variant surveillance, nucleic acid amplification tests (NAATs) such as reverse transcription polymerase chain reaction (RT‐PCR) have been explored.^11^, ^12^, ^13^, ^14^, ^15^, ^16^, ^17^, ^18^ The PCR‐based strategies differentiate among variants through the detection of unique, characteristic single‐nucleotide polymorphisms (SNPs). Several approaches demonstrate the SNP‐recognition capabilities inherent to PCR when primers or hydrolysis probes have single base mismatches that alter the quantification cycle (Cq) value at which amplification occurs for a target.^11^, ^17^ Since these variant detection platforms use common real‐time RT‐PCR practices, they are readily employable for use in most laboratory‐equipped settings. However, their reliance on slight changes in the efficiency of primer/probe binding makes these approaches susceptible to false negatives and positives.^19^ To enhance the specificity of RT‐PCR‐based approaches, the implementation of minor groove binding hydrolysis probes^13^ and locked nucleic acids^14^ have been proposed and evaluated. These approaches demonstrated high clinical performance for the majority of samples of Alpha, Beta, Gamma, and Delta variants but were limited to samples with high viral titers (e.g., Cq values ≤ 30 cycles). Melt‐analysis has also been coupled with RT‐PCR to determine the presence of SNPs by recognition of mismatch melt temperatures.^12^, ^16^ Although these studies demonstrated promising results, external quality assessments of the melt‐analysis and mutation‐specific PCR assays often rely on the detection of multiple variant sites and require relatively high viral load for accurate results.^19^, ^20^ Additionally, their performance is negatively impacted when additional polymorphisms are present.^19^, ^20^

For sequence‐free variant identification, we employ a ligation‐based molecular approach that classifies variants based on characteristic SNPs. The oligonucleotide ligation assay (OLA) is a highly specific nucleic acid hybridization‐based assay for the determination of SNPs.^21^ The assay discriminates single bases by joining two directly neighboring target recognition ligation probes if and only if the probes hybridize to the target at the bases nearest the junction. The OLA characteristically performs with high specificity and, when paired with NAATs, such as PCR, can achieve high sensitivity to detect low abundance SNPs in RNA and DNA targets.^22^, ^23^, ^24^, ^25^, ^26^, ^27^, ^28^

In this work, we present a generalizable method for the detection of known and future SARS‐CoV‐2 variants. The assay couples the OLA with a pre‐amplification RT‐PCR step and an endpoint real‐time PCR detection method to recognize characteristic SNPs for current VOCs. The analytical and clinical performances of the assay were evaluated to demonstrate its applicability to real‐world, population‐level testing. The general principles used in the development of the assay suggest that the method can be readily adapted for future emergent VOCs as well as other diseases. Overall, the work here yields a generalizable method for population‐level monitoring of known SARS‐CoV‐2 variants in settings where sequencing is either inaccessible or overwhelmed by high case numbers.

## 2 METHODS

### 2.1 Assay overview

**FIGURE 1:**
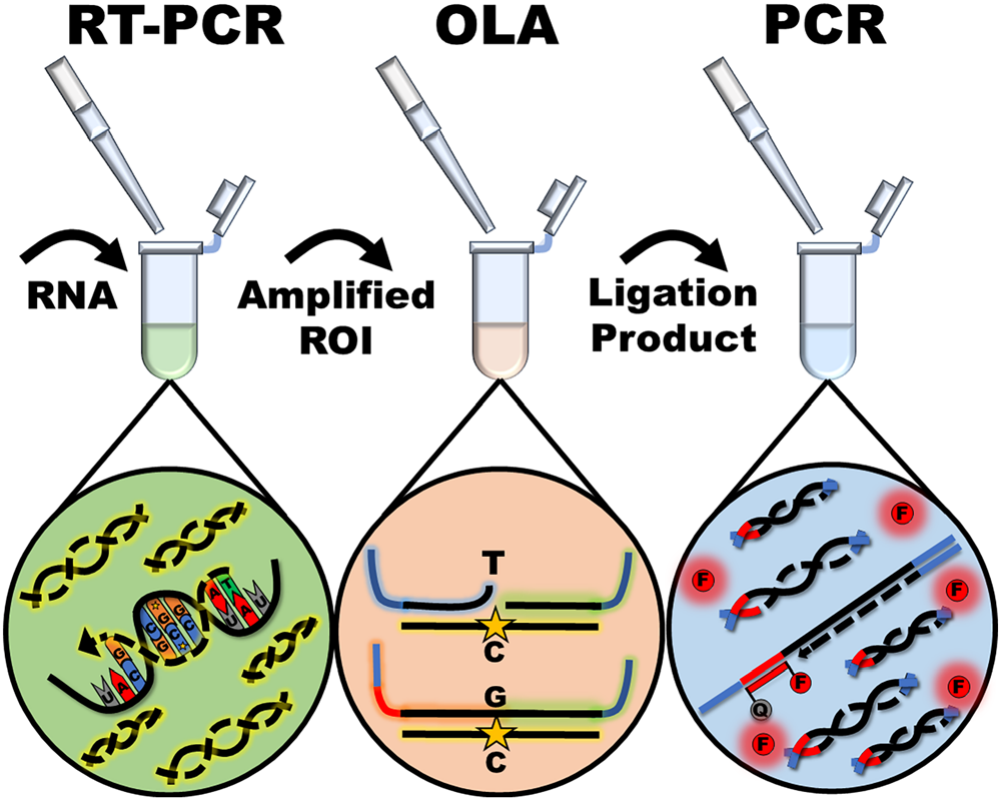
The ligation‐based genetic variant typing assay is composed of three sequentially conducted enzymatic assays. Regions of interest (ROIs) that contain single‐nucleotide markers of variants are first amplified by reverse transcription polymerase chain reaction (RT‐PCR). A portion of the RT‐PCR product is transferred to an oligonucleotide ligation assay (OLA) reaction where synthetic ligation probes are ligated into a single target if and only if hybridization occurs at the nucleotide of interest. A portion of the OLA product is then transferred to a real‐time PCR reaction for endpoint detection of ligated strands. The PCR signal indicates the presence of particular SNPs of interest.

The variant typing assay is designed to be generalizable for genetic surveillance of SNP patterns with clinical relevance. The assay sequentially links three enzymatic reactions for the amplification, discrimination, and detection of variant‐characteristic SNPs (Figure 1). Following RNA extraction, the assay is initiated by RT‐PCR to amplify regions of interest (ROIs) that contain the characteristic SNPs indicative of variant type or other clinically relevant features. The amplified ROIs are transferred to specific subsequent OLA reactions to investigate the sequences containing the characteristic SNPs.

The OLA discriminates between nucleotides through a hybridization‐based ligation event. The ligation occurs if and only if the 3′ end of a synthetic ligation probe hybridizes to the single nucleotide of interest. By introducing multiple oligonucleotide ligation probes with alternate 3′ nucleotides, known as variable probes (VPs), the ligation event, which occurs with a singular, always‐binding common probe (CP), achieves very high specificity. Since the ligation probes are synthetically manufactured, specific and exogenous primer and hydrolysis probe binding sites can be concatenated to the ligation probes for real‐time PCR detection in the following reaction.

**FIGURE 2:**
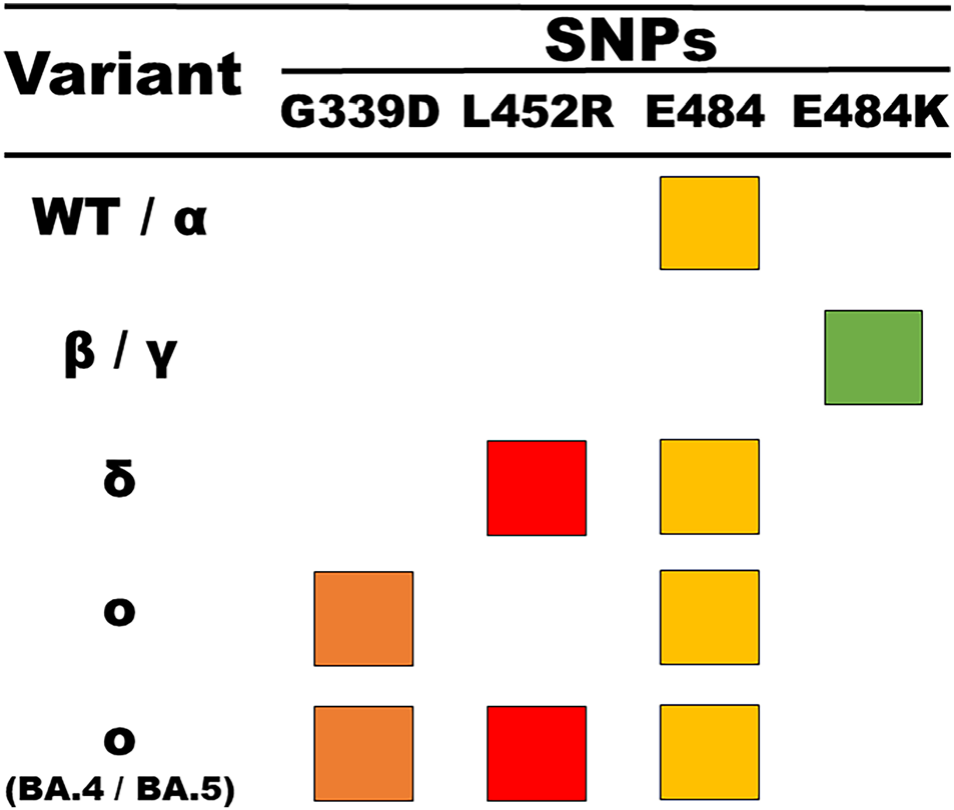
SARS‐CoV‐2 genetic variants are determined by designing discriminating patterns across four fluorescence channels of real‐time PCR signals generated from the ligation‐based assay. The assay investigates three SNPs of interest, G339D, L452R, and E484K, as well as one Alpha/positive control marker E484. Variant PCR signals and their respective fluorescence channel are indicated by row and color.

PCR is used to sensitively and specifically amplify transferred ligation product as a marker of the presence of SNPs of interest. Endpoint PCR was selected as the detection modality since a thermal cycler was already a pre‐requisite of the assay for pre‐OLA amplification, the platform is highly specific, and the additional amplification step provides extra sensitivity to aid in the detection of low viral load samples (Figure S1). The PCR reaction employs exogenous primers and probes to enhance the specificity of the assay. Signals from each PCR reaction are used to determine the sample variant type in combination according to an SNP‐based typing chart (Figure 2).

In this SARS‐CoV‐2‐specific implementation of the variant detection assay, there are two ROIs that are amplified from the receptor binding domain (RBD) within the spike gene. The RBD was selected because of the abundance of genetic mutations in each of the VOCs and its likely continued accumulation of mutations in future VOCs. The two ROIs encapsulate (1) amino acid (AA) 339 and (2) AAs 452 and 484. These regions contain mutations unique to Omicron (G339D), Delta (L452R), and Beta/Gamma (E484K) as well as a marker for a positive control indicative of the wild‐type sequence (E484). At the time of assay development, L452R was unique to Delta; however, the emergent sublineages of Omicron, BA.4 and BA.5, contain this SNP. In these cases, the assay differentiates between Delta and Omicron variants as BA.4 and BA.5 contain the G339D marker such that proper classification requires both signals. The SNP‐based variant typing chart for this application is presented in Figure 2. In this work, two workflows were conducted in the evaluation of the assay. The first workflow tests for different SNP markers by performing each enzymatic reaction in a singleplex, whereas the second workflow combines each enzymatic reaction into a multiplex workflow (Figure S0). The first workflow is presented to demonstrate the most generalizable approach to adapting this method, and the second workflow demonstrates the multiplexable nature of the assay to reduce the technical burden of assay performance.

### 2.2 Design/rules for OLA‐based genetic typing

**FIGURE 3:**
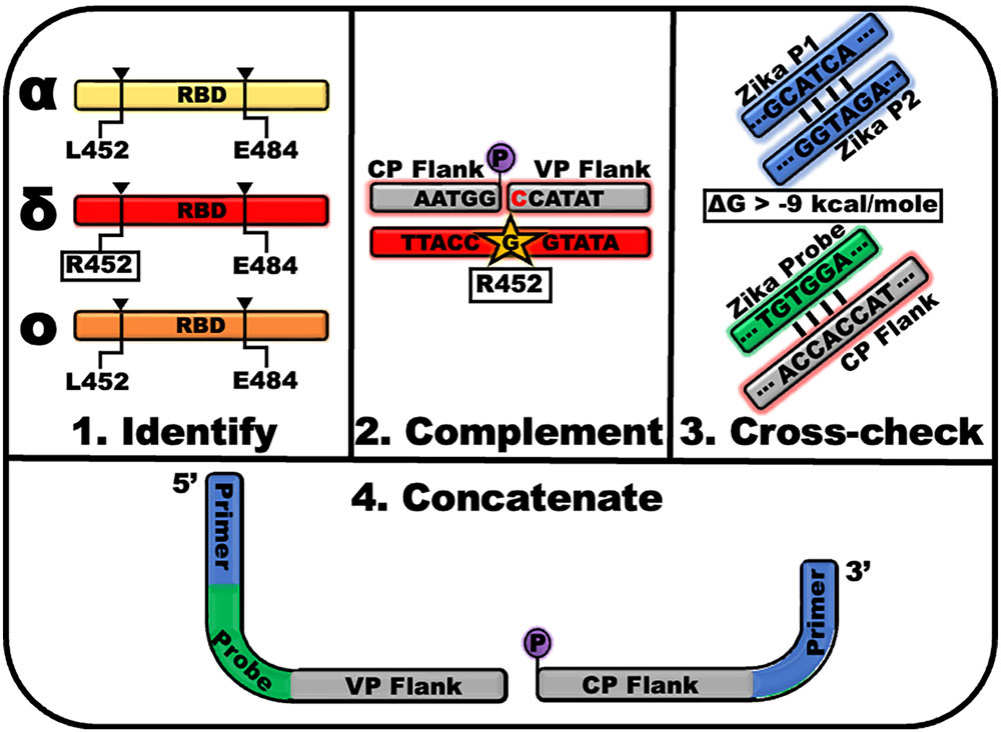
Design of the oligonucleotide ligation assay (OLA) reaction for genetic variant typing is conducted with four essential steps. (1) Identification of single‐nucleotide markers that are unique to the variant or disease state of interest. (2) Creation of the common and variable ligation probe, CP and VP respectively, hybridization region by complementing the target strand in the sense that will be present in the OLA reaction. The length of each flanking region is determined by accounting for the desired melt temperature. (3) Investigation of the hybridization or cross‐reactivity of the exogenous primers and hydrolysis probes that will be used for downstream real‐time detection as well as the ligation probe flanking regions that will be in the OLA reaction. (4) Concatenation of the primer and flanking regions for each ligation probe as well as the hydrolysis probe binding region for the SNP‐discriminatory VP.

The variant detection assay is generalizable for genetic typing of variant sublineages, future emerging variants, and for other diseases that can be classified by SNP patterns. The key components of the variant detection assay design process can be described in four primary steps: (1) identification of the characteristic SNP, (2) design of the ligation probe SNP‐flanking regions, (3) design and addition of primer probe sites, and (4) concatenation and evaluation of the ligation probes (Figure 3).

Identification of the characteristic SNPs is essential for discriminating variants by the ligation‐based assay (Figure 3, Panel 1). Characteristic SNPs are single‐base change patterns that occur uniquely and consistently in the variant or sublineage of interest. The tool is not realistically applicable to the discovery of new variants or newly emergent SNPs; therefore, the differentiating SNP patterns must be identified by existing known sequences. As sequencing data are collected, consensus sequences are generated and the one or more consistent mutations that differentiate an emerging variant from others are determined based on a set of determined nomenclature rules.^29^ To employ this assay, it is assumed that new variants are classified prior to selecting the SNPs of interest. The mutations that were used to classify new variants are the characteristic SNPs that should be targeted in this assay design. As the target of interest, in this case, SARS‐CoV‐2, evolves, some SNPs may be signaled in other variants. In this study, the Delta discriminating SNP evolved in later sublineages of Omicron. These cases rely on information from multiple channels to differentiate with continued success (e.g., G339D positive or negative signal may distinguish Delta from Omicron). Also, multiple SNPs may be present in the sequences for differentiation, in which case one is selected and evaluated for its simulated performance, described in Step 3, and its experimental performance. Reselection of the SNP of interest may be necessary following the results of the proceeding steps. An example of a ligation set that needed to be reselected is shown in Figure S2. However, many additional tools aid in the characteristic SNP identification process; some of which provide lists of variant‐specific single‐nucleotide mutations that are useful for ready adaptation of the ligation‐based assay presented here.^30^, ^31^, ^32^, ^33^

Once the single‐nucleotide mutation of interest is selected, the target‐hybridizing regions of the ligation probes are designed (Figure 3, Panel 2). These ligation probe “flanking regions” consist of the target‐complementary sequences on both sides of the SNP of interest. In the context of DNA or following the RT‐PCR amplification step, the complementary sequences could be designed for either sense (positive or negative) of the target. If only an RT step is used prior to the OLA, then the flanking regions must be designed to hybridize the complementary DNA, which will have the opposite sense of the RNA target. This can be leveraged to optimize the melting temperature by shifting the frame of the target sequence in the case of ligation probes of differing lengths. The length of the ligation probe flanking regions can vary from 15 bases up to 30 bases.^23^, ^34^, ^35^ However, in general, the greater the length of the flanking region, the greater the tolerance for additional surrounding polymorphisms. The flanking region complementary to the sequence downstream of the SNP serves as the basis for the variable ligation probe (VP). The VP also contains the base complementary to the single nucleotide of interest at its 3′ end. An additional VP containing the wild‐type base at the point mutation is ideal to include in the OLA reaction to induce competition‐based specificity. The flanking region complementary to the target upstream of the SNP serves as the basis for the CP. The 5′ end of the CP, the end nearest the SNP of interest, must be modified with a phosphate for the OLA reaction to occur. The ligation assay works most specifically when the melt temperatures of the VPs and the CP are within a few degrees Centigrade of one another. Designing the ligation probes for the probe length, sense, and melt temperatures is greatly aided using ligase manufacturer‐provided tools such as NEB's Ligase Calculator.^36^ In this design, the HiFi *Taq* Ligase is utilized, which requires the VP to hybridize with the sequence downstream of the SNP of interest. However, other ligases enable the VP to hybridize either upstream or downstream of the mutation, which may affect the fidelity of the SNP discrimination. Additionally, alternative ligase enzymes may have varied dependence on other non‐synonymous mutations near the SNP of interest that alters the enzyme efficiency and specificity.^35^ If alternative ligases are desirable for others' intended use, these effects should be considered in the assay design.

For the ligation‐based variant assay to yield the greatest specificity, detection of the ligated product by real‐time PCR should be achieved using primers and hydrolysis probes with sequences that are not likely to be found in the clinical sample. In this work, primers and probes were selected for infectious diseases that are unlikely to co‐infect a COVID‐19 patient in the region of our study as these PCR reactions had been developed for previous projects (e.g., Zika Virus). In practice, the background target is not likely to cause a false signal because of the employment of highly specific sequential reactions. Alternative schemes that intentionally mismatch the primer and probe targets may also be used in this modular assay design. The highly specific primer binding sites are included in both the VPs and the CP, and the hydrolysis binding sites are included in the SNP‐recognition VP; therefore, it is essential that the sequences of all primers, probes, and flanking regions be cross‐checked for their individual and combined binding affinity (Figure 3, Panel 3). High binding affinity (ΔG < approximately −9 kcal/mol), as measured by Gibbs' free energy (ΔG), can result in dimerization that causes either false ligation in the OLA reaction or false priming events in the endpoint PCR reaction and compromise the specificity of the variant detection platform (Figure S2). Spurious ligation events can occur when the ligation probes non‐specifically hybridize to a nucleic acid in the ligation reaction at the ligation junction (Figure S2). For the PCR reaction, any dimerization of the primers will lower the reaction efficiency. Yet, most consequentially, a false priming event by a nucleic acid binding at its 3′‐end to the variable ligation probe may induce a false signal by enabling the amplification of a target containing the hydrolysis probe binding region. OligoAnalyzer, a free tool provided by Integrated DNA Technologies (IDT), assists in the determination of self and heterodimers that may occur between the oligonucleotides used in the variant detection assay.^37^ For non‐specific binding caused by the hydrolysis probe or primers, those sequences can be simply switched to another primer/probe set that is unlikely to be in the sample type. In the case of non‐specific hybridization events relating exclusively to the ligation probe hybridization regions, the characteristic SNP should be changed to another unique mutation with ligation flanking regions that are not susceptible to generating a false signal if possible. When a multiplex reaction is desired, it is desirable to perform this cross‐checking step between all oligonucleotide components contained in the combined OLA reaction as there is a non‐zero possibility that, for example, one ligation probe hybridizes with the junction of another ligation set and induces a non‐specific ligation event.

**FIGURE 4:**
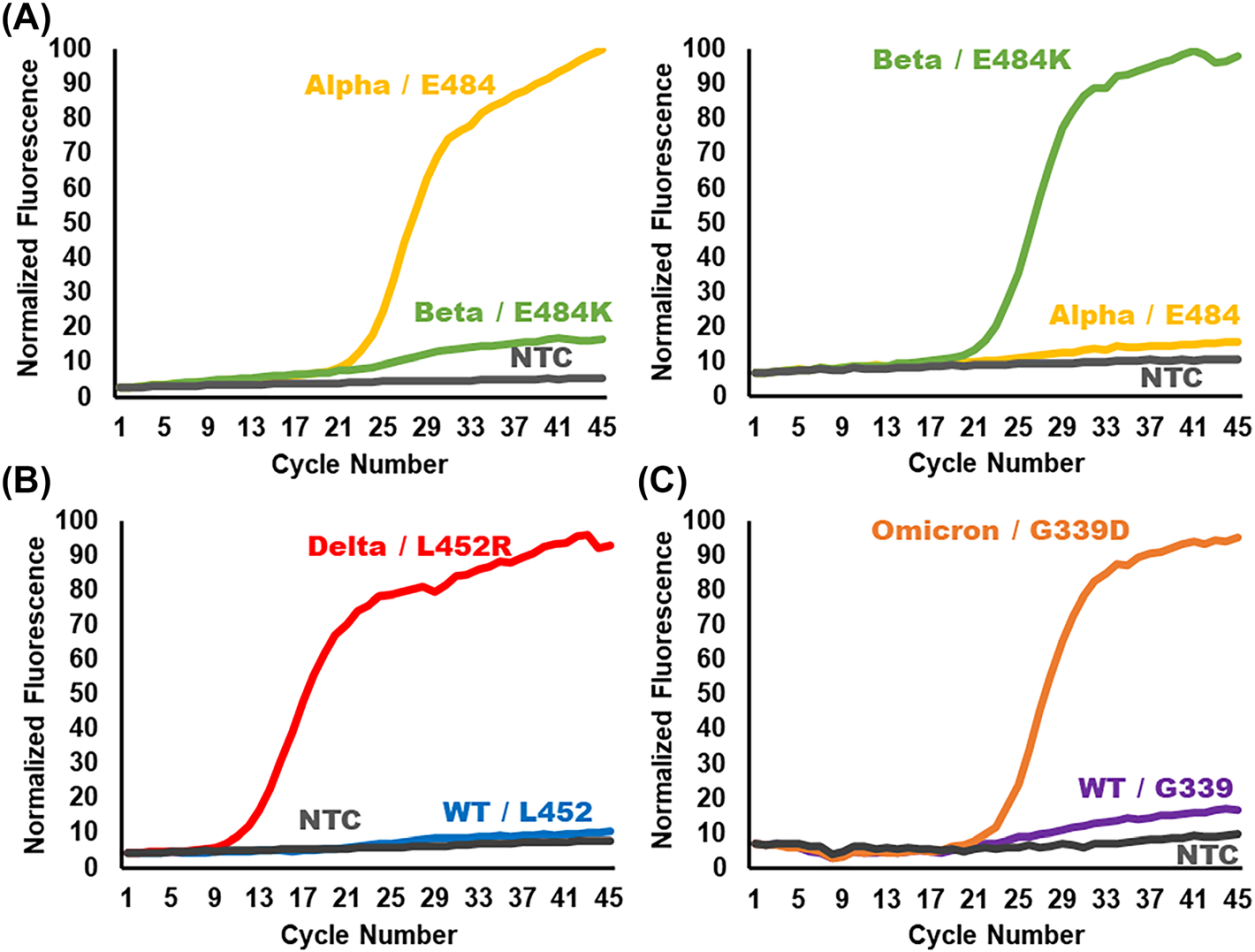
Ligation‐based variant typing produced a highly specific signal for the detection of unique single‐nucleotide polymorphisms (SNPs) in synthetic SARS‐CoV‐2 DNA targets or RT‐PCR products of purified genomic RNA from BEI resources. (A) The Alpha and Beta single‐nucleotide markers, E484 (yellow) and E484K (green), are performed in a single multiplexed OLA reaction without cross‐reactivity for the detection of 10^6^ copies of synthetic DNA targets. The multiplexed E484(K) ligation set amplifies in all COVID‐19‐positive samples and, therefore, serves as a sample control for the assay. (B) Singleplex OLA for the L452R marker (red), characteristic of the Delta variant, specifically detects the mutation without non‐specific amplification of the wild‐type L452 marker (blue) for the RT‐PCR product of Delta variant purified genomic RNA. (C) The G339D mutation (orange) is detected as a characteristic marker of the Omicron variant. The ligation reaction for G339D does not produce amplified signal for the wild‐type G339 marker (purple). The G339D ligation pair was evaluated on 10^6^ copies of synthetic DNA target. The representative data show desired performance of ligation reactions in the screening process.

The final step in designing the ligation probes for the variant detection platform is the concatenation of the primer sites, hydrolysis probe sites, and the flanking regions (Figure 3, Panel 4). The mutation‐detection VP consists of, from 5′ to 3′, the sequence matching an exogenous primer followed by a “CGC” DNA linker, the hydrolysis probe binding site, the flanking region complementary to the downstream target sequence, and the SNP‐complementary base. The DNA linker provides a space between the primer and probe binding site to maximize the efficiency of the PCR reaction. The competitive WT VP uses the same form without the hydrolysis probe binding site. The CP consists of a phosphate, the flanking region complementary to the target sequence upstream of the SNP, and the complementary binding site for the other exogenous primer. Prior to the deployment of new ligation probes, the probes should be tested on known sequences for their efficacy and specificity. Validation of the ligation probes designed to detect the Alpha, Beta/Gamma, Delta, and Omicron VOCs was performed on either genomic RNA of known variants or synthetic oligonucleotides mimicking the sequence of the variant target (Figure 4). These data show no amplification of off‐target sequences nor amplification of no template controls (NTCs) and, therefore, demonstrate the high analytical specificity of the OLA reaction employed in the following studies. Additionally, these data serve as an example for the design and development of future adaptations of the ligation‐based genetic typing assay. A flow chart to replicate the design process is provided in the Supporting Information (Figure S6).

### 2.3 Sample extraction

Contrived and clinical COVID‐19‐positive samples were extracted by spin columns from the QIAamp Viral RNA Mini Kit (Qiagen #52906). Samples were added to spin columns at a volume of 140 μl and eluted in a final volume of 60 μl of Qiagen AVE buffer.

### 2.4 Variant assay

The variant detection assay is intended for use with known COVID‐19‐positive samples. It was designed to detect four major VOCs that have emerged throughout the course of the pandemic: Alpha (B.1.1.7/Q.x), Beta (B.1.351)/Gamma (P.1) (paired), Delta (B.1.617.2/AY.x), and Omicron (BA.x). The assay was developed for testing in the United States where the Beta and Gamma variants were not known to circulate, and thus, the assay was designed to combine these two variants into a single output. The variant assay workflow was initiated with pre‐amplification on extracted samples by RT‐PCR. Product from the RT‐PCR reaction was directly pipetted into the OLA for SNP discrimination. Detection of the ligated targets was performed by real‐time PCR following direct pipette transfer of a fraction of the OLA product (Figure 1). Each step of the assay is outlined in the following methods section. All synthetic oligonucleotides including primers, hydrolysis probes, and ligation probes were synthesized by IDT. The composition of each reaction in the variant typing assay is tabulated for reproducibility in the Supporting Information (Tables S1–S3).

### 2.5 Pre‐amplification (RT‐PCR)

RT‐PCR was conducted for the initial amplification of SARS‐CoV‐2 ROIs surrounding variant‐characterizing SNPs. The two ROIs encompassed (1) AA 339 and (2) AAs 452 and 484 for the detection of Omicron and detection of Alpha/Beta and Delta, respectively. RT‐PCR was performed with the SensiFAST™ SYBR® No‐ROX One‐Step Kit (Meridian Bioscience, BIO‐72005). The RT‐PCR reaction was composed of 1× SensiFAST™ master mix (10 μl), 1× RNase inhibitor (0.4 μl), ROI‐specific primers at a final concentration of 400 nM, 6 μl of SARS‐CoV‐2 viral extract, 1× reverse transcriptase (0.2 μl), and molecular grade water to bring the reaction to 20 μl total. The pre‐amplification step was performed with a Rotor‐Gene Q (Qiagen, Germantown, MD). Reverse transcription was performed at 45°C for 15 min and was immediately followed by a 95°C hold for 2 min. Three‐step PCR amplification was conducted by 40 cycles of 95°C for 5 s, 60°C for 20 s, and 72°C for 5 s. Green fluorescence data were collected at the end of the 72°C extension step. For low copy/high Cq targets, as quantified by the FDA‐approved COVID‐19 RT‐PCR assays, or samples with high levels of interferent, an initial 95°C heat step for 1 min was implemented prior to the addition of the reverse transcriptase to promote target‐primer interaction. Primers for the pre‐amplification RT‐PCR are summarized in Table 1. Pre‐amplified targets were transferred to the subsequent reaction immediately or stored at 4°C until ready for use.

**TABLE 1:** Oligonucleotide sequences designed for the SARS‐CoV‐2 variant typing assay

Description	Sequence (5′ ➔ 3′)
RT‐PCR AA339 ROI F Primer	GCTGTAGACTGTGCACTTGA
RT‐PCR AA339 ROI R Primer	TGCTGATTCTCTTCCTGTTCC
RT‐PCR AA452 + 484 ROI F Primer	AGGCTGCGTTATAGCTTGGA
RT‐PCR AA452 + 484 ROI R Primer	GTTGGAAACCATATGATTGTAAAGG
G339(D) Ligation Common Probe	/5Phos/TGAAGTTTTTAACGCCACCAGATTTG ‐ TTCAGCAGAGAGAATTCACTCAG
G339 WT Ligation Variable Probe	CAAAAGGAAGTCGCGCAATA ‐ CGC ‐ ATATTACAAACTTGTGCCCTTTTG **G**
G339D MUT Ligation Variable Probe	CAAAAGGAAGTCGCGCAATA ‐ CGC ‐ GCGCGCTCCCAGCCACATG ‐ ATATTACAAACTTGTGCCCTTTTG **A**
G339(D) PCR F Primer (Dengue)	CAAAAGGAAGTCGCGCAATA
G339(D) PCR R Primer (Dengue)	CTGAGTGAATTCTCTCTGCTGAAC
G339D PCR Hydrolysis Probe	/5TEX615/CATGTGGCTGGGAGCGCGC/3IAbRQSp/
L452(R) Ligation Common Probe	/5Phos/GGTAATTATAATTACCACCAACCTTAGGAGAAAAAGATGGGACAGGTG
L452 WT Ligation Variable Probe	CAGCTGGCATCATGAAGAACC ‐ CGC ‐ TTAGACTTCCTAAACAATCTATAC **A**
L452R MUT Ligation Variable Probe	CAGCTGGCATCATGAAGAACC ‐ CGC ‐ CCACTATTCCATCCACAACGG ‐ TTAGACTTCCTAAACAATCTATAC **C**
L452(R) PCR F Primer (Zika)	CAGCTGGCATCATGAAGAACC
L452(R) PCR R Primer (Zika)	CACCTGTCCCATCTTTTTCTCC
L452R PCR Hydrolysis Probe	/5Cy5/CCGTTGTGG /TAO/ATGGAATAGTGG/3IAbRQSp/
E484(K) Ligation Common Probe	/5Phos/AAGGTTTTAATTGTTACTTTCCTTTA ‐ TTATGAGAAATCAAAGTCTTTGGGTT
E484 WT Ligation Variable Probe	GTTAAGGGAGTGAAGACGATCAGA ‐ GCG ‐ CTAGTCGGCATAGTTTATGGTTAAGATTACGACGGT ‐ GGTAGCACACCTTGTAATGGTGTT **G**
E484K MUT Ligation Variable Probe	GTTAAGGGAGTGAAGACGATCAGA ‐ GCG ‐ AGTCATCTTTCGAGGTGACTTTTAGATTGCT ‐ GGTAGCACACCTTGTAATGGTGTT **A**
E484(K) PCR F Primer	GTTAAGGGAGTGAAGACGATCAGA
E484(K) PCR R Primer	AACCCAAAGACTTTGATTTCTCATAA
E484 WT PCR Hydrolysis Probe	/5HEX/ ACCGTCGTA/ZEN/ATCTTAACCATAAACTATGCCGACTAG/3IABkFQ/
E484K MUT PCR Hydrolysis Probe	/56‐FAM/ AGCAATCTA/ZEN/AAAGTCACCTCGAAAGATGACT/3IABkFQ/

*Note*: Both wild‐type and mutant bases that are discriminated by the oligonucleotide ligation assay for variant typing are marked in red.

### 2.6 Oligonucleotide ligation assay

Following the pre‐amplification step, 10% (2 μl) of the RT‐PCR product was transferred into its SNP‐specific OLA reaction. Three SNP‐specific OLA reactions targeting E484/E484K, L452R, and G339D were employed for the detection of the four variant groups aforementioned. The RT‐PCR product encompassing AA339 was added to the G339D OLA reaction, and the product containing AA452 and AA484 was transferred to the L452R and E484/E484K OLA reactions. The ligation assay was conducted with the high‐fidelity, thermostable HiFi *Taq* DNA Ligase (NEB #M0647). Reactions were performed with 0.2 μl of the HiFi *Taq* Ligase and 2 μl of the 10× HiFi *Taq* Ligase Buffer. Three ligation probes, two VPs with different 3′ nucleotides, and one CP, specific for each SNP of interest, were added to their respective OLA reaction. The CP was added to have a final concentration of 1 nM. The VPs were added to have final concentrations of 10 nM, with the exception of the E484/E484K ligation set in which the VPs were at a final concentration of 1 nM to reduce cross‐over noise that was occurring in the reaction. This cross‐over reduction was observed by empirical study (data not shown). Molecular‐grade water was added to bring the reaction to 20 μl total. The reaction was performed with a 95°C hold for 1 min followed by a 60°C hold for 10 min. The ligation probe sequences are reported in Table 1.

### 2.7 Endpoint real‐time PCR

Real‐time PCR was used to detect ligated probes via ligation‐product‐specific primers and hydrolysis probes. PCR reactions were performed using the Luna Universal Probe qPCR Kit (NEB #M3004). Each reaction contained 10 μl of the Luna Universal Probe Master Mix and 0.4 μl of each of the SNP‐specific primers and probes for a final concentration of 200 nM. Ten percent (2 μl) of the OLA product was transferred directly into the PCR reaction. Molecular‐grade, nuclease‐free water was added to the reaction to bring the final volume to 20 μl. Following an initial 3‐min hold at 95°C, two‐step PCR cycling was performed at 95°C for 15 s and 60°C for 15 s. Fluorescence was measured at the end of the 60°C annealing/extension step. The PCR reaction was performed with a Rotor‐Gene Q (Qiagen, Germantown, MD), and analysis was performed with the analysis feature of the Rotor‐Gene Q Series software (version 2.0.3). The Cq quantification threshold was standardized across all experiments where Cq was analyzed.

### 2.8 Multiplex variant assay

The variant assay was also converted to a multiplexed format to enhance the ease of assay performance. Pre‐amplification RT‐PCR was conducted by including both primer sets for the two ROIs. Each primer was added at a final concentration of 400 nM. Cycling and kit conditions were conserved from the singleplex format. The multiplex OLA was performed with 2 μl of multiplexed RT‐PCR product in the presence of all four SNP‐specific sets of variable and common ligation probes at a final concentration of 1 nM. The reaction conditions and heating were conserved from the singleplex OLA reaction. Two microliters of multiplexed OLA product were transferred to the multiplex endpoint PCR reaction. Cycling conditions were conserved with the exception of shortening the number of cycles to 35 to eliminate late amplification noise from reagent cross‐over reactivity. The endpoint PCR reaction was performed with all primers and probes at a final concentration of 200 nM.

### 2.9 Limit of detection

The variant assay limit of detection (LOD) was determined by extracting and evaluating contrived COVID‐19‐positive samples. The contrived samples consisted of pooled negative nasopharyngeal matrix collected at Vanderbilt University Medical Center (VUMC) spiked with pre‐classified gamma‐irradiated virus from BEI Resources. Each variant type of gamma‐irradiated virus was spiked into the pooled matrix at final concentrations of 10^2^ genome copies/μL and 10^1^ copies/μL prior to extraction and variant assay performance. Twenty independent samples of each concentration for each variant type were evaluated. The LOD was determined to be the target concentration at which 19 of 20 samples were detected in the endpoint real‐time PCR of the variant detection assay. Limits of detection were identified specifically for each variant type. The following reagents were obtained through BEI Resources, NIAID, NIH: SARS‐Related Coronavirus 2, Isolate USA‐WA1/2020, Gamma‐Irradiated, NR‐52287, contributed by the Centers for Disease Control and Prevention; SARS‐Related Coronavirus 2, Isolate hCoV‐19/USA/MD‐HP01542/2021 (Lineage B.1.351), in *Homo sapiens* Lung Adenocarcinoma (Calu‐3) Cells, Gamma‐Irradiated, NR‐55351, contributed by Andrew S. Pekosz; SARS‐Related Coronavirus 2, Isolate hCoV‐19/USA/MD‐HP05285/2021 (Lineage B.1.617.2; Delta Variant), Gamma‐Irradiated, NR 56129, contributed by Andrew S. Pekosz; and SARS‐Related Coronavirus 2, Isolate hCoV‐19/USA/GA‐EHC‐2811C/2021 (Lineage B.1.1.529; Omicron Variant), Gamma‐Irradiated, NR‐56496, contributed by Mehul Suthar.

### 2.10 Clinical study design

Residual, derivative‐of‐care SARS‐CoV‐2‐positive specimens (nasal swabs in viral transport media) were obtained from the VUMC Diagnostic Laboratories, under the approval of the International Review Board (IRB #201708 and #201804). The samples were aliquoted and stored at −80°C between original COVID‐19 testing and further analyses for this study. Although not part of the original reportable diagnostic result, the Cq for study specimens was determined on the FDA‐authorized Panther Fusion qRT‐PCR system (Hologic, Marlborough, MA). Absolute quantification of viral burden was facilitated here via the inclusion of quantitative calibrators, traceable to International Unit (IU) standards of the World Health Organization. Predicate determination of viral clade was made by Illumina next‐generation sequencing (NGS). RNA was extracted from clinical specimens using the vRNA Mini kit (Qiagen), and libraries were prepared with the QIAseq DIRECT SARS‐CoV‐2 with QIAseq DIRECT SARS‐CoV‐2 Enhancer kit. Sequencing was performed on a NovaSeq with paired 150‐bp reads. Viral assembly and variant assignment were conducted with Cecret (https://github.com/UPHL-BioNGS/Cecret ) SARS‐CoV‐2 workflow (v.2.1.2021117.1), with resultant sequence information deposited in GISAID.^38^

The results of this sequencing were blinded from the investigators conducting the ligation‐based variant assay. Some samples were evaluated under double‐blinded conditions by the performance of the ligation‐based variant assay prior to NGS. In cases where classified positive samples did not have sufficient volume for extraction and quantitation, samples were diluted in viral transport media prior to quantitation and variant assay testing to ensure that the concentration of the sample was consistent between evaluation platforms.

### 2.11 Analysis

Analysis and estimations of the variant‐specific LOD studies were performed by linear regression of logarithmically plotted sample viral load. Cq estimations for the LOD studies were determined by comparison with absolute quantitation data provided by the inclusion of known viral load calibrators in the Panther RT‐PCR assay. Statistical analyses, including variant‐specific population percentages and significance testing between viral load data sets, were performed in MATLAB R2020a. Statistical significance was determined by two‐sample *t*‐testing (*α* = 0.05). Clinical sequence alignment and analysis were performed with MegAlign Pro sequence alignment software (DNASTAR, Madison, WI).

## 3 RESULTS

### 3.1 Analytical sensitivity

**FIGURE 5:**
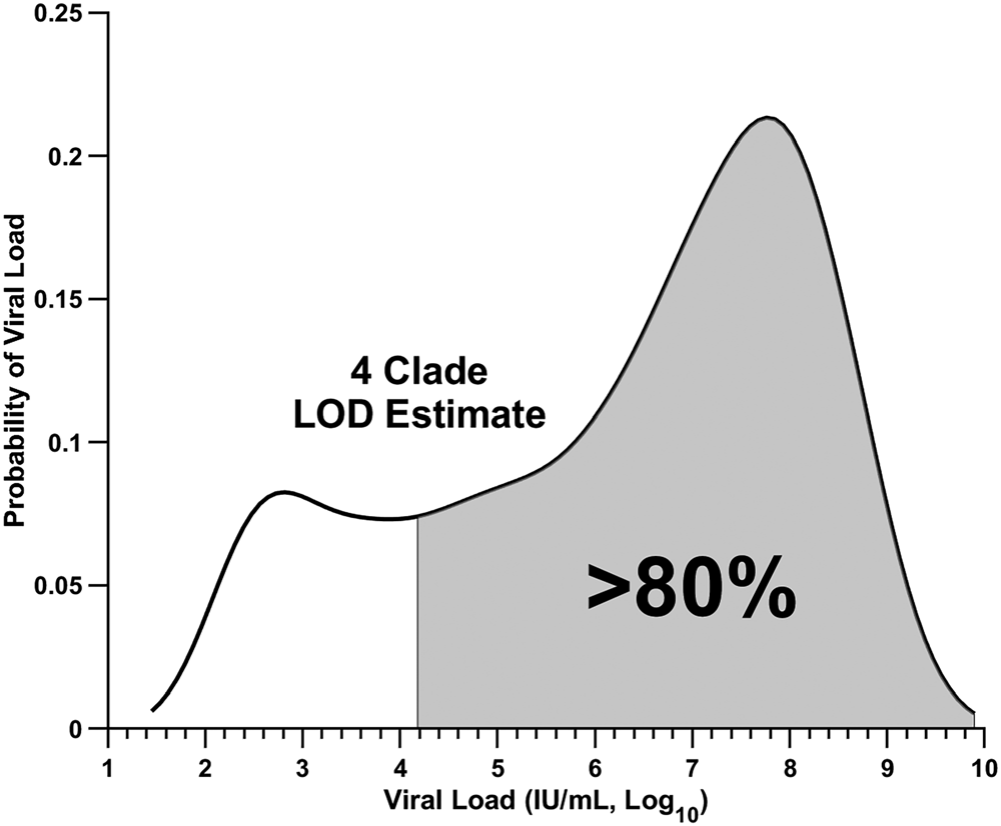
The variant detection assay is estimated to provide sequencer‐free classification in a majority of positive COVID‐19 samples. For each variant, the limit of detection was determined by evaluation of contrived samples containing inactivated virus in clinical pooled negative nasopharyngeal matrix. The detection limits were converted to an estimated viral load (IU/mL) to determine the percentage of samples detectable from the COVID‐19 population‐level data. An estimated 80.5% of positive COVID‐19 samples (shaded) are expected to be detectable by all four clades by the variant typing assay. Estimation is based on the probability density function of sample viral load from population‐level, untyped clinical COVID‐19 samples (black line). The population‐level data set was adapted from Victoriano et al.^39^

To determine the analytical performance of the variant detection assay, the LOD was evaluated for each variant. Contrived samples consisting of gamma‐irradiated SARS‐CoV‐2 virus spiked into COVID‐19‐negative pooled clinical nasopharyngeal matrix were extracted and evaluated by the ligation‐based variant detection platform. For each variant, 20 independent contrived samples for each concentration neighboring the detection limits (10^5^ and 10^4^ genome copies/mL) were tested to determine the capabilities of the assay. The assay consistently detected at least 19 of 20 (≥95%) contrived samples at the 10^5^ copies/mL concentration for each variant (Table 2). Linear approximations of each variant LOD were 10^5^, 10^4^, 8.6 × 10^4^, and 6.3 × 10^4^ copies/mL for Alpha, Beta, Delta, and Omicron, respectively (Figure S3). These values correspond to approximately 79.4%, 87.8%, 80.1%, and 81.1% of the population based on a distribution of viral load values attained from 5160 COVID‐19 samples collected (Figure S4). The population data set was adapted from Victoriano et al.^39^ Overall, the assay is estimated to detect approximately 7.4 × 10^4^ copies/mL, which corresponds to an estimated 80.5% of the entire population of untyped clinical nasopharyngeal samples (Figure 5). Therefore, COVID‐19‐positive samples with this viral load or higher are expected to be detected at least 95% of the time.

**TABLE 2:** Summary of the analytical performance of the ligation‐based variant typing assay

Variant (SNP)	Samples detected at 10^4^ copies/mL	Samples detected at 10^5^ copies/mL	Interpolated LOD estimate (copies/mL)
Alpha (E484)	16/20	19/20	1 × 10^5^
Beta (E484K)	20/20	20/20	1 × 10^4^
Delta (L452R)	5/20	20/20	8.6 × 10^4^
Omicron (G339D)	15/20	20/20	6.3 × 10^4^
Total	56/80	79/80	7.4 × 10^4^

*Note*: Limit of detection (LOD) was estimated by logarithmic regression to determine the 95% hit rate. The LOD studies were performed on contrived COVID‐19 samples consisting of known‐variant inactivated virus spiked into pooled negative clinical nasopharyngeal matrix. The percentage of clinical samples detectable by the variant assay was estimated through a comparative analysis between the analytical performance of the assay and the population viral load data collected at VUMC.

### 3.2 Clinical sensitivity and specificity

Residual de‐identified clinical nasopharyngeal samples were extracted and evaluated by the ligation‐based variant assay. For 88 known positive COVID‐19 samples, the assay achieved 96.6% sensitivity (95% binomial proportion confidence interval [CI]: 90.4%–99.3%) and 99.5% specificity (95% CI: 97.3%–99.99%) in blinded clinical trials across all variant types collected (Table 3). Each sample was evaluated by the variant assay for their typing and compared with their sequencing results (Table S4). Alpha variant samples were identified with 100% sensitivity (95% CI: 88.1%–100%) and 100% specificity (95% CI: 94.8%–100%). The variant assay identified Delta samples with 90% sensitivity (95% CI: 73.5%–97.9%) and 100% sensitivity (95% CI: 94.7%–100%). Omicron samples were identified by the variant assay with 100% sensitivity (95% CI: 88.1%–100%) and 98.6% specificity (95% confidence interval: 92.2%–99.96%).

**TABLE 3:** Summary of the clinical performance of the ligation‐based variant detection assay evaluating extracted nasopharyngeal COVID‐19 patient samples

Variant	True positive	True negative	False positive	False negative	Average viral load (IU/mL, Log_10_)	Sensitivity (%)	Specificity (%)
Alpha	29	69	0	0	5.58	100	100
Delta	27	68	0	3	6.47	90	100
Omicron	29	68	1	0	6.42	100	98.6
Total	85	205	1	3	6.18	96.6	99.5

*Note*: Next‐generation sequencing was used as the gold standard for accuracy determination. Viral load per variant type was calculated based on quantified internal calibrators (*n* = 88 positive COVID‐19 samples, 10 independent trials).

**FIGURE 6:**
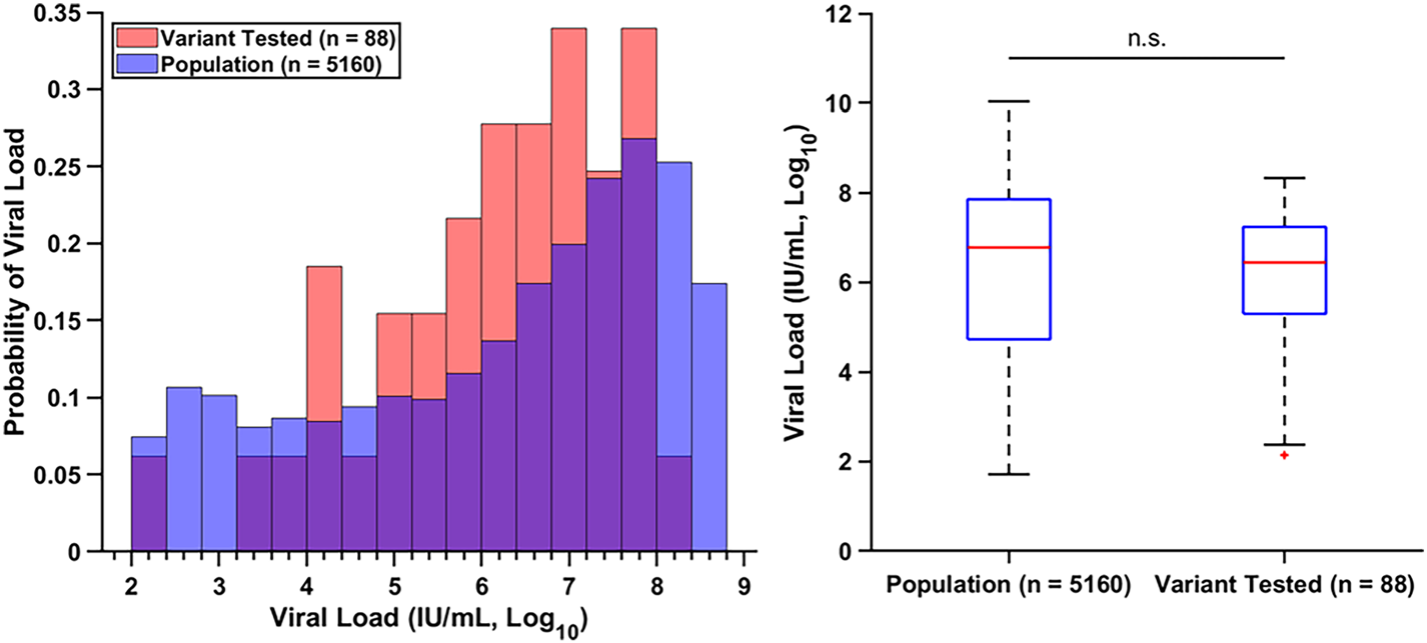
The positive COVID‐19 samples evaluated by the ligation‐based variant typing assay were representative of the population samples with respect to viral load. The probability density histograms (left) of sample viral load from standard COVID‐19 RT‐PCR between the samples evaluated by the typing assay (red, *n* = 88) and samples collected from the population (blue, *n* = 5160) show a large overlap (purple) between the two sample groups. Boxplots of the sample groups (right) demonstrate the range, 25th and 75th percentiles, and averages of each group. The sample groups were not significantly different from each other as determined by a two‐sample *t*‐test (*p* = 0.787, *α* = 0.05). The population‐level data set was adapted from Victoriano et al. ^39^

To ensure the robustness of the study, the viral load distribution of the clinical samples investigated in this study was compared with the population‐level viral load distribution from samples collected at VUMC from March 2020 to March 2021 (*n* = 5160) (Figure 6). The study sample viral load distribution was not found to be statistically different from the population viral load distribution.

### 3.3 Multiplex assay performance

Following singleplex clinical trials, multiplexing of the variant assay was investigated to reduce the number of assay steps required to conduct the assay. In the multiplexed format, the three sequential enzymatic reactions are each performed as individual multiplex reactions, which enabled a simple three‐tube workflow as compared with the eight individual reactions required for full VOC screening in the singleplex format. The multiplex assay detected all samples with Cq values of <28.2 (4.29 Log_10_ IU/mL), which corresponds to approximately 79% of the population based on the population data set (Table S5). The multiplexed format demonstrated an overall concordance with a singleplex of 98.6%, accounting for all true positives and negatives based on the singleplex reaction as the ground truth. Compared with NGS, the multiplexed format had an overall clinical sensitivity and specificity of 93.2% (CI: 85.7%–97.5%) and 99.0% (CI: 96.4%–99.9%), respectively (Table 4).

**TABLE 4:** Summary of the clinical performance of the ligation‐based variant detection assay in the multiplexed format

Variant	True positive	True negative	False positive	False negative	Sensitivity (%)	Specificity (%)	Concordance with Singleplex (%)
Alpha	25	66	0	4	86.2	100	95.8
Delta	29	64	1	1	96.7	98.5	97.8
Omicron	28	65	1	1	96.6	98.5	98.9
**Total**	82	195	2	6	93.2	99.0	98.6

*Note*: The multiplexed assay was performed on the same extracted nasopharyngeal COVID‐19 patient samples as singleplex testing. Sample identification accuracy was determined by comparison with next‐generation sequencing. Concordance was determined by the summation of true positive and negatives divided by the total number of samples in which singleplex results were considered the ground truth (*n* = 88 positive COVID‐19 samples, 7 independent trials).

### 3.4 Ligation performance with polymorphisms in the hybridization region

**FIGURE 7:**
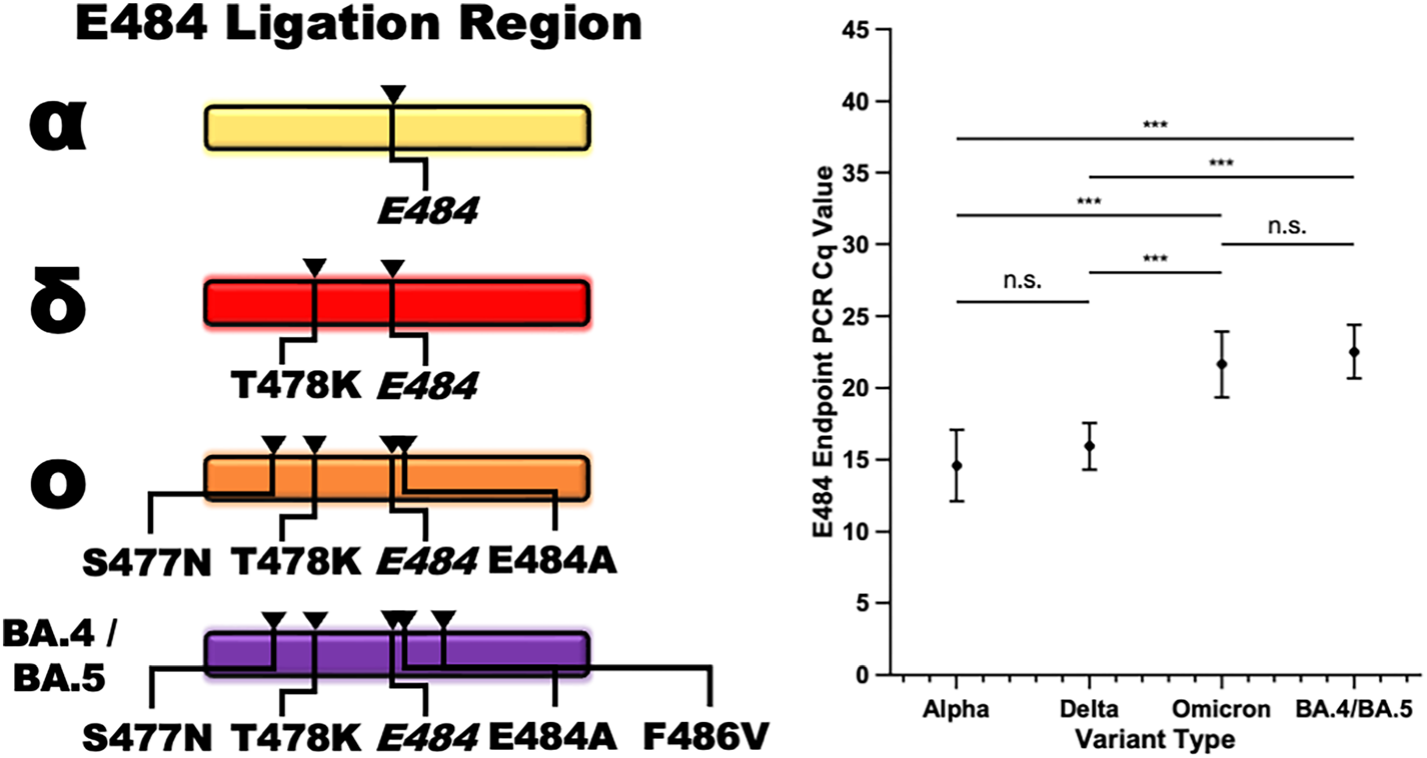
The ligation‐based variant detection assay performed robustly in the presence of surrounding non‐synonymous single‐nucleotide polymorphisms (SNPs). The Delta and Omicron variants of concern developed SNPs in the ligation probe hybridization region of the ligation control/Alpha detection ligation set (left). In clinical samples, the presence of a single polymorphism in the hybridization region, found in the Delta samples, did not significantly alter the performance of the assay (right). The presence of multiple polymorphisms, most importantly E484A which occurs at the base immediately neighboring the base affected by the E484(K) mutation, as found in the Omicron clinical samples, resulted in a significant delay in endpoint PCR detection (paired two‐sample *t*‐tests with Bonferroni correction for four groups, *α* = 0.05. *p < 0.05, **p < 0.025, ***p < 0.01).

Clinical sample sequences were aligned with the ligation target regions of each variant. The target region for the E484 ligation set was subjected to an additional non‐synonymous SNP, namely, T478K, in the ligation probe hybridization region for Delta‐classified variants. The presence of the extra SNP did not induce a significant change in the assay performance as measured by Cq in the endpoint PCR reaction (Figure 7). Similarly, the E484 ligation set was subject to surrounding polymorphisms in all Omicron samples. Three polymorphisms, namely, S477N, T478K, and E484A, were consistently located within the E484 flanking ligation regions in all Omicron samples. In all cases and counts, including the presence of the E484A mutation, which affects hybridization on the 5′ end of the CP, the surrounding polymorphisms did not impede ligation events when the marker of interest was present although it did delay the endpoint PCR amplification significantly for Omicron samples as compared with Alpha samples (*p* = 3 × 10^−15^, two‐sample *t*‐test) and Delta samples (*p* = 4.1 × 10^−18^, two‐sample *t*‐test) (Figure 7).

## 4 DISCUSSION

This work demonstrates a generalizable, sequencer‐free variant typing assay applied for detecting known variants of SARS‐CoV‐2. The assay was performed with high sensitivity and specificity in both clinical and contrived COVID‐19 samples. The assay was performed with a clinical sensitivity of 96.6% and specificity of 99.5% across all sample types. The misclassifications in the study occurred when evaluating low viral load samples only in the Delta channel, which also had the worst LOD in contrived sample testing. The assay also correctly identified the variant in at least 95% of contrived samples with a viral load of approximately 7.4 × 10^4^ genome equivalents/mL, which maps to an estimated 80.5% of historically collected, untyped positive COVID‐19 samples from the general population and approximately 85% of all symptomatic cases (Figure S5). The contrived samples consist of pooled negative COVID‐19 viral transport matrix, which is intended to maximize the potential diversity of background interferents. Together, these studies suggest that the variant typing assay will work robustly in COVID‐19 samples even with relatively high levels of background interferent. By achieving robust detection of >95% even at low viral loads in clinical samples, the assay surpasses the CDC recommendations for sequencing samples with Cq ≤ 28, suggesting that the sequencer‐free variant assay is at least as good as the gold standard for typing known variants.^40^ The multiplexed format of the variant detection assay achieved detection of all samples with Cq < 28.2 in clinical studies and, therefore, is likely to be similarly useful as the gold‐standard NGS per the CDC recommendations even with the slight loss of sensitivity. Additionally, the sequencer‐free variant assay presented in this work is inexpensive and more accessible in comparison with NGS. As such, evaluation of lower viral load samples that may not yield guaranteed detection is not as resource‐prohibitive and may yet produce valuable variant classification information.

A major benefit of the demonstrated approach is the generalizability of the assay. In this work, a guide to reproducing and extending the principles of the ligation‐based variant typing assay is provided. Additionally, a design flow chart is provided in the Supporting Information. By following the four relatively simple steps presented, the assay can be adapted to future emergent SARS‐CoV‐2 variants or other diseases with single‐nucleotide markers of interest. Although this work does not show the adaptation of the assay to other diseases, the principle is demonstrated by the previous employment of the OLA to other applications including HIV drug resistance,^23^, ^24^, ^26^, ^27^, ^28^, ^41^ pancreatic cancer,^22^ and tuberculosis,^42^ among others. Additionally, aside from the custom oligonucleotides necessary for the generalized use of the assay, the variant typing assay employs off‐the‐shelf RT, PCR, and ligation reagents enabling relatively simple preparation.

The variant typing assay was performed effectively in the presence of additional base mutations surrounding the SNPs of interest. Tolerance of the surrounding polymorphisms was demonstrated most thoroughly in the hybridization regions of the E484 ligation pair for Delta and Omicron samples. In particular, the assay successfully detected the E484 SNP in Omicron samples, which are known to include a non‐synonymous base change, E484A, immediately neighboring the SNP of interest as well as at two to three other SNPs in the ligation probe hybridization region. When the E484A mutation was present, there was a significant delay in the endpoint PCR signal. However, because of the pre‐ligation amplification step, the target signal remained high enough to tolerate the inhibition. The delayed signal is still amplified early enough to likely tolerate an even higher density of surrounding polymorphisms. The tolerance of neighboring polymorphisms is a product of the robustness of the oligonucleotide ligation assay.^27^, ^28^, ^43^ This suggests that the assay would be useful in high mutation variants where primer‐based or melt‐analysis variant detection strategies may be subject to false signals. Additionally, these studies suggest that developed ligation sets are likely sustainable for detection as organisms evolve over time.

The current iteration of the ligation‐based variant typing assay could be enhanced by the inclusion of automated processing. The assay requires manual processing to transfer products through the sequential enzymatic reactions, which can be burdensome on the operator and increase the risk of laboratory contamination from opening tubes containing RT‐PCR amplicons. Future automation by strategies such as magnetic processing or robotic liquid handling through a self‐contained platform would minimize contamination and technical burden.^44^, ^45^, ^46^ Optimizing the workflow to enable testing of unextracted samples would further improve the processing burden.^39^ The ligation‐based assay may also benefit from adopting alternative amplification and detection strategies that do not require a real‐time PCR instrument. However, in its current state, since the assay employs real‐time PCR for endpoint detection of variants, the utilization of the RT‐PCR as the pre‐amplification enables operators to use the same equipment. Additionally, the high sensitivity of the endpoint PCR step enhances the capabilities of the assay to detect low viral load samples. Yet, overcoming the need for a PCR device would further improve the accessibility for the generalizable genetic‐typing assay.

Overall, the work presented demonstrates a sequencer‐free genetic variant typing assay that robustly detects known variants of SARS‐CoV‐2 in a manner that is sustainable through the evolution of the virus. The ligation‐based assay is readily adaptable to future SARS‐CoV‐2 variants and to other diseases for the detection of characteristic SNPs or other single bases of interest. Although the approach presented in this work cannot replace the discovery capabilities of genetic sequencing, the variant typing assay provides a promising means for expanding access to SARS‐CoV‐2 variant monitoring and detection.

## CONFLICTS OF INTEREST

There are no conflicts of interest to declare.

## AUTHOR CONTRIBUTIONS

**Dalton J. Nelson:** Conceptualization; data curation; formal analysis; investigation; methodology; project administration; validation; writing‐original draft. **Meghan H. Shilts:** Data curation; formal analysis; investigation; methodology; writing‐review and editing. **Suman B. Pakala:** Data curation; formal analysis; investigation; methodology; writing‐review and editing. **Suman R. Das:** Data curation; funding acquisition; project administration; resources; writing‐review and editing. **Jonathan E. Schmitz:** Conceptualization; funding acquisition; investigation; methodology; project administration; resources; supervision; writing‐review and editing. **Frederick R. Haselton:** Conceptualization; funding acquisition; methodology; project administration; resources; supervision; writing‐original draft; writing‐review and editing.

## ETHICS STATEMENT

All specimens were collected, de‐identified, and transferred from Vanderbilt University Medical Center as approved by the Institutional Review Board (IRB #201708 and #201804).

### PEER REVIEW

The peer review history for this article is available at https://publons.com/publon/10.1111/irv.13083.

## Supporting information


**Figure S0.** Overview of the two separate workflows for the variant assay. The singleplex workflow (top) separated the RT‐PCR reactions by the targeted region of interest, and subsequent reactions were performed in separate pathways for each contained variant‐specific SNPs. The singleplex workflow is the most modular and readily adaptable format for future applications. The ligation‐based assay is also highly multiplexable (bottom). By multiplexing the workflow, the assay workflow is decreased to just three pipette transfers and the technical burden for conducting the assay is reduced.
**Figure S1.** The endpoint PCR detection strategy enables detection of ligated products of low viral load SARS‐CoV‐2 samples. Most samples with higher viral load (> ~ 3 Log_10_ IUs / mL) result in a very low variant‐specific endpoint PCR quantification cycle (Cq) (~12–17) due to the pre‐ligation amplification step by RT‐PCR. However, as shown by representative samples DN57 (red) and DN58 (orange) with viral loads of 2.13 and 2.37 Log_10_ IUs/mL, respectively, low viral load samples are detected at late cycles. These results are still clearly differentiable from No Target Controls (NTC, gray dotted) and, therefore, can still be interpreted as positive signals.
**Figure S2.** Representative PCR curves showing false signal generated by self‐hybridizing ligation probes. Ligation probes designed to recognize the Q498R spike mutation presented in the Omicron variant were susceptible to spurious ligation events that resulted in false positive signals in endpoint PCR when no target was present in the ligation reaction. Spurious ligation likely occurred due to a hybridization event that bridged the junction (red arrow) of the variable and common ligation probes, as shown by the OligoAnalyzer results (top). While the ligation reaction is highly specific, these events need to be screened during the variant‐typing assay design process.
**Figure S3.** Estimations of the variant‐typing assay limits of detection for each variant of concern. Approximations were based on a logarithmic‐linear regression from experimental data presented in **Table 2**. The limit of detection was determined as the viral load for which 95% or more samples are detectable. For each variant, contrived samples were generated by spiking known‐variant inactivated virus into pooled negative nasopharyngeal matrix. Extracts from contrived samples were tested by the variant‐typing assay to determine the analytical sensitivity. Two concentrations were evaluated for each variant surrounding the limit of detection and each concentration was evaluated with n = 20 independent samples.
**Figure S4.** Estimations of the variant‐typing assay detection performance for untyped, population‐level positive COVID‐19 clinical samples for each variant‐specific platform. Approximations were based on the limit of detection determined by a logarithmic‐linear regression from experimental data presented in **Table 2**. The percentage of samples detectable per variant‐specific ligation platform (shaded) was determined by comparison to the probability distribution function of the untyped, historically collected clinical samples (black line, n = 5160).
**Figure S5.** Estimations of the variant‐typing assay detection performance for untyped, population‐level positive COVID‐19 clinical samples for all COVID‐19 positive cases and symptomatic COVID‐19 cases. The variant‐typing assay is estimated to detect ~85% of all symptomatic cases (bottom) for all four clades investigated in this work. For all COVID‐19 samples, including asymptomatic and test for cure patients, the assay is anticipated to detect approximately 80% of samples.
**Figure S6.** Flow chart depicting the design process for the oligonucleotide ligation assay that serves as the crux of the variant‐typing assay. The design theory is generalizable for other diseases marked by characteristic single nucleotide polymorphisms by conducting a series of design and decision steps (left). In practice, not all pathways are followed, and some iteration will likely be necessary as shown in the example for the Omicron variant‐specific detection of the G339D mutation (center). Unfollowed pathways are marked by dotted lines to match the theory. For each step along the design and decision workflow, a combination of different tools and platforms were employed for this specific application to SARS‐CoV‐2 variants (right). These tools can be used for extension to future SARS‐CoV‐2 variants, and the general tools can be used to apply this strategy to other diseases. The tools are horizontally aligned with the steps in which they are relevant.
**Table S1.** Experimental setup for the pre‐ligation amplification step by RT‐PCR using the SensiFAST SYBR No‐Rox One‐Step kit. Total volume used for each RT‐PCR reaction was 20 μl.
**Table S2.** Experimental setup for the singleplex oligonucleotide ligation assay using the HiFi Taq DNA Ligase. The total volume of the reaction is 20 μl. The reaction was conducted on 10% of the RT‐PCR product containing amplicons of the region of interest containing the characteristic SNP. For the E484 and E484K combined ligation reaction, the mutant and wild‐type probe final concentrations were adjusted to 1 nM.
**Table S3.** Experimental setup for the endpoint PCR reaction following the oligonucleotide ligation assay. The total volume for the PCR reaction is 20 μl. †The wild‐type/competing SNP hydrolysis probe may not be used in all cases. For our application, this was only employed in the duplexed E484/E484(K) assay. If a hydrolysis probe is not used, the volume for the probe should be replaced with molecular grade water as denoted in the table.
Table S4. Summary of clinical trials with the singleplex variant‐typing assay.
**Table S5. Summary of clinical trials with the multiplex variant‐typing assay.** Multiplex trials were performed following the singleplex trials, therefore, known negative samples were not performed on the multiplex platform as indicated by black boxes.

## Data Availability

Data associated with this study is available in the main text and Supplementary Information. Additional data, including raw PCR data files and sequence FASTA files, will be made available at 10.5281/zenodo.7036209 for reviewers and to the public following publication.
