# Supplementary material for: Ligation‐based assay for variant typing without sequencing: Application to SARS‐CoV‐2 variants of concern
Source: Influenza Other Respir Viruses. 2022 Dec 12;17(1):e13083. doi: 10.1111/irv.13083 (PMC9835417; doi:10.1111/irv.13083)
Supplement: Supplementary file 1 — Figure S0. Overview of the two separate workflows for the variant assay. The singleplex workflow (top) separated the RT‐PCR reactions by the targeted region of interest, and subsequent reactions were performed in separate pathways for each contained variant‐specific SNPs. The singleplex workflow is the most modular and readily adaptable format for future applications. The ligation‐based assay is also highly multiplexable (bottom). By multiplexing the workflow, the assay workflow is decreased to just three pipette transfers and the technical burden for conducting the assay is reduced. Figure S1. The endpoint PCR detection strategy enables detection of ligated products of low viral load SARS‐CoV‐2 samples. Most samples with higher viral load (> ~ 3 Log10 IUs / mL) result in a very low variant‐specific endpoint PCR quantification cycle (Cq) (~12–17) due to the pre‐ligation amplification step by RT‐PCR. However, as shown by representative samples DN57 (red) and DN58 (orange) with viral loads of 2.13 and 2.37 Log10 IUs/mL, respectively, low viral load samples are detected at late cycles. These results are still clearly differentiable from No Target Controls (NTC, gray dotted) and, therefore, can still be interpreted as positive signals. Figure S2. Representative PCR curves showing false signal generated by self‐hybridizing ligation probes. Ligation probes designed to recognize the Q498R spike mutation presented in the Omicron variant were susceptible to spurious ligation events that resulted in false positive signals in endpoint PCR when no target was present in the ligation reaction. Spurious ligation likely occurred due to a hybridization event that bridged the junction (red arrow) of the variable and common ligation probes, as shown by the OligoAnalyzer results (top). While the ligation reaction is highly specific, these events need to be screened during the variant‐typing assay design process. Figure S3. Estimations of the variant‐typing assay limits of detection for [file IRV-17-0-s001.docx]

**Supplementary Materials**

**Ligation-based Assay for Variant-Typing without Sequencing: Application to SARS-CoV-2 Variants of Concern**

Dalton J. Nelson^1^, Megan H. Shilts^2^, Suman B. Pakala^2^, Suman R. Das^2,4^, Jonathan E. Schmitz^3,4^, and Frederick R. Haselton^1,^*

^1^ Department of Biomedical Engineering, Vanderbilt University, Nashville, Tennessee, United States

^2^ Department of Medicine, Vanderbilt University Medical Center, Nashville, Tennessee, United States

^3^ Department of Pathology, Microbiology and Immunology, Vanderbilt University Medical Center, Nashville, Tennessee, United States

^4^ Vanderbilt Institute for Infection, Immunology and Inflammation, Vanderbilt University Medical Center, Nashville, Tennessee, United States

* To whom correspondence should be addressed. Tel: (615)-322-6622; Fax: (615)-343-7919; Email: [Rick.Haselton@vanderbilt.edu](mailto:Rick.Haselton@vanderbilt.edu)

**Contents**

[**Ligation-based Variant Assay Workflows** 2](#_Toc120020910)

[**Variant Assay Reactions Setup** 3](#_Toc120020911)

[**Low Viral Load Detection with Endpoint PCR** 4](#_Toc120020912)

[**Ligation Target False Signal** 4](#_Toc120020913)

[**Limit of Detection Estimations** 5](#_Toc120020914)

[**Variant-Specific Detection Estimates** 6](#_Toc120020915)

[**Symptomatic Case Variant-Assay Estimate** 7](#_Toc120020916)

[**Clinical Sample Viral Load Calculations** 7](#_Toc120020917)

[**Variant-Assay Design Flow Chart** 8](#_Toc120020918)

[**Singleplex Clinical Results** 9](#_Toc120020919)

[**Multiplex Clinical Results** 12](#_Toc120020920)

# **Ligation-based Variant Assay Workflows**


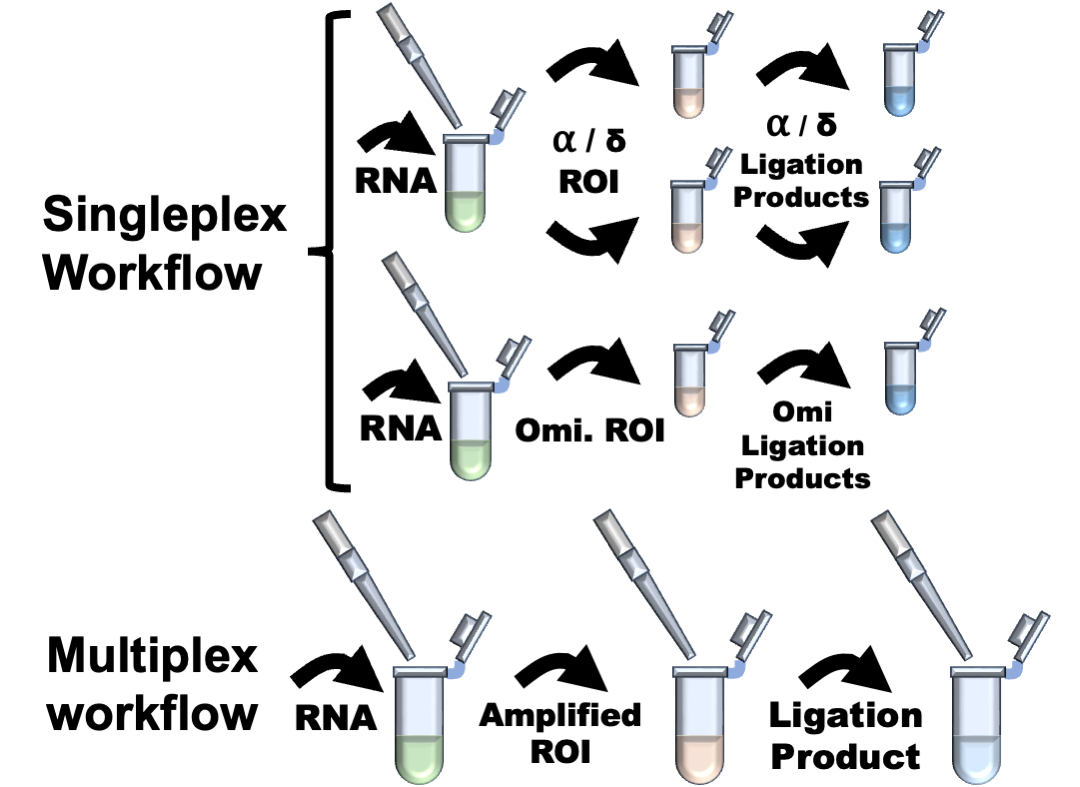


**Figure S0. Overview of the two separate workflows for the variant assay.** The singleplex workflow (top) separated the RT-PCR reactions by the targeted region of interest, and subsequent reactions were performed in separate pathways for each contained variant-specific SNPs. The singleplex workflow is the most modular and readily adaptable format for future applications. The ligation-based assay is also highly multiplexable (bottom). By multiplexing the workflow, the assay workflow is decreased to just three pipette transfers and the technical burden for conducting the assay is reduced.

# **Variant Assay Reactions Setup**

*RT-PCR*

| **Component** | **[Stock]** | **[Final]** | **Volume/Reaction (µL)** |
| --- | --- | --- | --- |
| Molecular Grade Water | - | - | 1.8 |
| SensiFAST One-Step Mix | 2x | 1x | 10 |
| Reverse Transcriptase | 100x | 1x | 0.2 |
| RNase Inhibitor | 50x | 1x | 0.4 |
| Forward Primer | 10µM | 400nM | 0.8 |
| Reverse Primer | 10µM | 400nM | 0.8 |
| Extracted RNA Target | - | - | 6 |

**Table S1.** Experimental setup for the pre-ligation amplification step by RT-PCR using the SensiFAST SYBR No-Rox One-Step kit. Total volume used for each RT-PCR reaction was 20µL.

*Oligonucleotide Ligation Assay*

| **Component** | **[Stock]** | **[Final]** | **Volume/Reaction (µL)** |
| --- | --- | --- | --- |
| Molecular Grade Water | - | - | 13.4 |
| HiFi Taq Ligation Buffer | 10x | 1x | 2 |
| HiFi Taq Ligase | 1 reaction/µL | 5 reaction/µL | 0.2 |
| Wild-type Variable Probe | 1µM | 10nM | 0.2 |
| Mutant Variable Probe | 1µM | 10nM | 0.2 |
| Common Ligation Probe | 10nM | 1nM | 2 |
| RT-PCR Product | - | - | 2 |

**Table S2.** Experimental setup for the singleplex oligonucleotide ligation assay using the HiFi Taq DNA Ligase. The total volume of the reaction is 20µL. The reaction was conducted on 10% of the RT-PCR product containing amplicons of the region of interest containing the characteristic SNP. For the E484 and E484K combined ligation reaction, the mutant and wild-type probe final concentrations were adjusted to 1nM.

*Endpoint PCR*

| **Component** | **[Stock]** | **[Final]** | **Volume/Reaction (µL)** |
| --- | --- | --- | --- |
| Molecular Grade Water | - | - | 6.8 (6.4†) |
| Luna Universal Probe Mix | 2x | 1x | 10 |
| Forward Primer | 10µM | 200nM | 0.4 |
| Reverse Primer | 10µM | 200nM | 0.4 |
| SNP of Interest Hydrolysis Probe | 10µM | 200nM | 0.4 |
| WT / Competing SNP Hydrolysis Probe† | 10µM | 200nM | 0.4 |
| Ligation Product | - | - | 2 |

**Table S3.** Experimental setup for the endpoint PCR reaction following the oligonucleotide ligation assay. The total volume for the PCR reaction is 20µL. †The wild-type / competing SNP hydrolysis probe may not be used in all cases. For our application, this was only employed in the duplexed E484 / E484(K) assay. If a hydrolysis probe is not used, the volume for the probe should be replaced with molecular grade water as denoted in the table.

# **Low Viral Load Detection with Endpoint PCR**


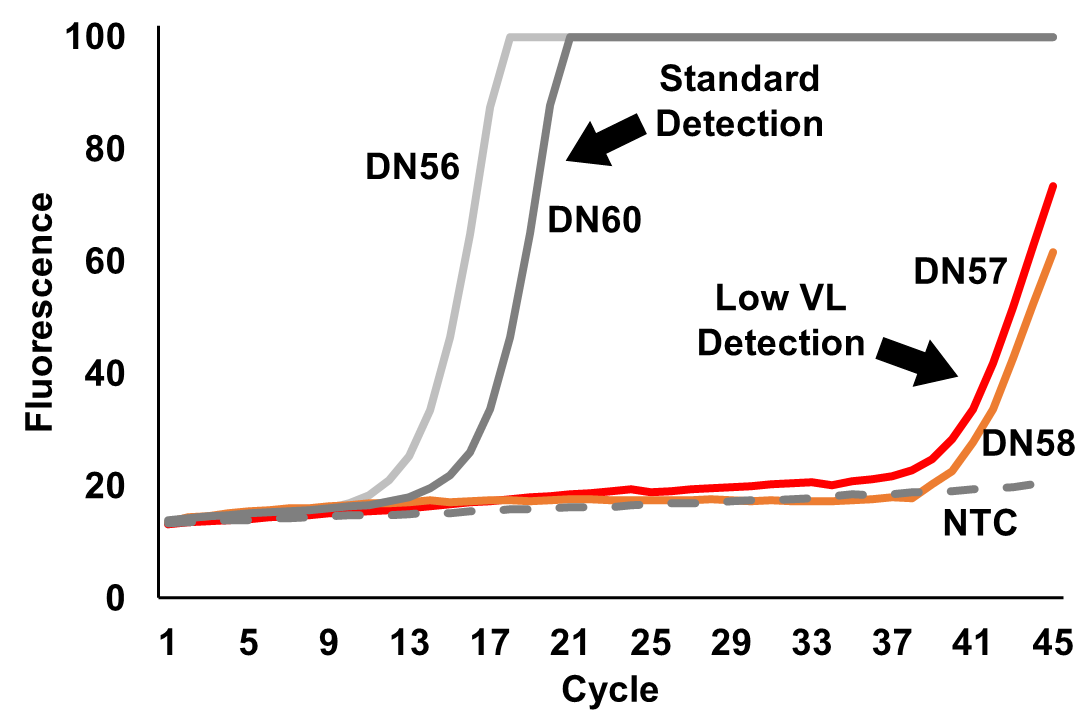


**Figure S1.** The endpoint PCR detection strategy enables detection of ligated products of low viral load SARS-CoV-2 samples. Most samples with higher viral load (> ~3 Log_10_ IUs / mL) result in a very low variant-specific endpoint PCR quantification cycle (Cq) (~12-17) due to the pre-ligation amplification step by RT-PCR. However, as shown by representative samples DN57 (red) and DN58 (orange) with viral loads of 2.13 and 2.37 Log_10_ IUs / mL, respectively, low viral load samples are detected at late cycles. These results are still clearly differentiable from No Target Controls (NTC, gray dotted) and, therefore, can still be interpreted as positive signals.

# **Ligation Target False Signal**


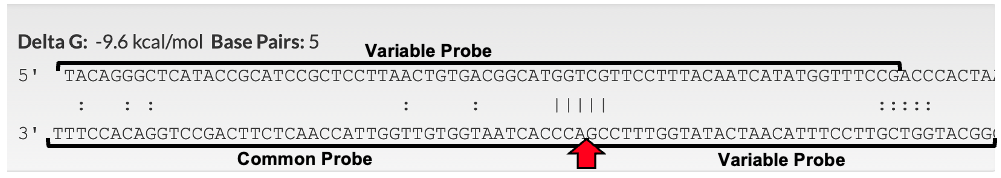

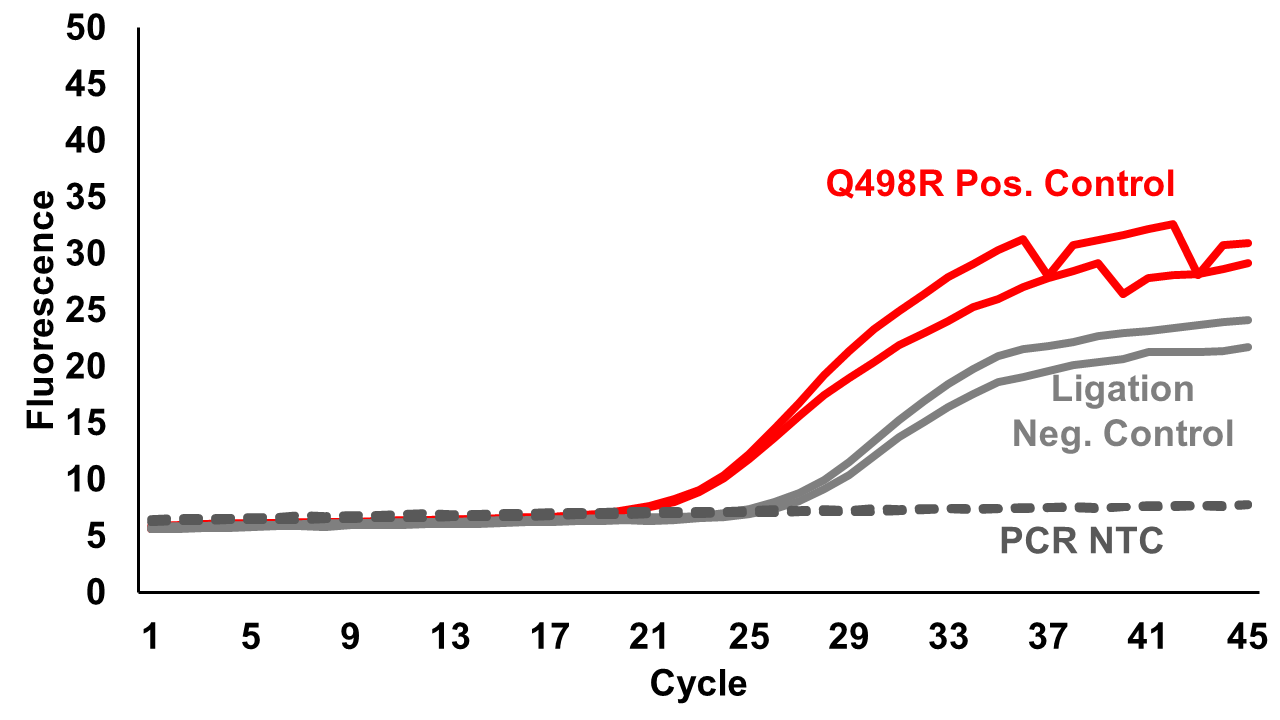


**Figure S2.** Representative PCR curves showing false signal generated by self-hybridizing ligation probes. Ligation probes designed to recognize the Q498R spike mutation presented in the Omicron variant were susceptible to spurious ligation events that resulted in false positive signals in endpoint PCR when no target was present in the ligation reaction. Spurious ligation likely occurred due to a hybridization event that bridged the junction (red arrow) of the variable and common ligation probes, as shown by the OligoAnalyzer results (top). While the ligation reaction is highly specific, these events need to be screened during the variant-typing assay design process.

# **Limit of Detection Estimations**


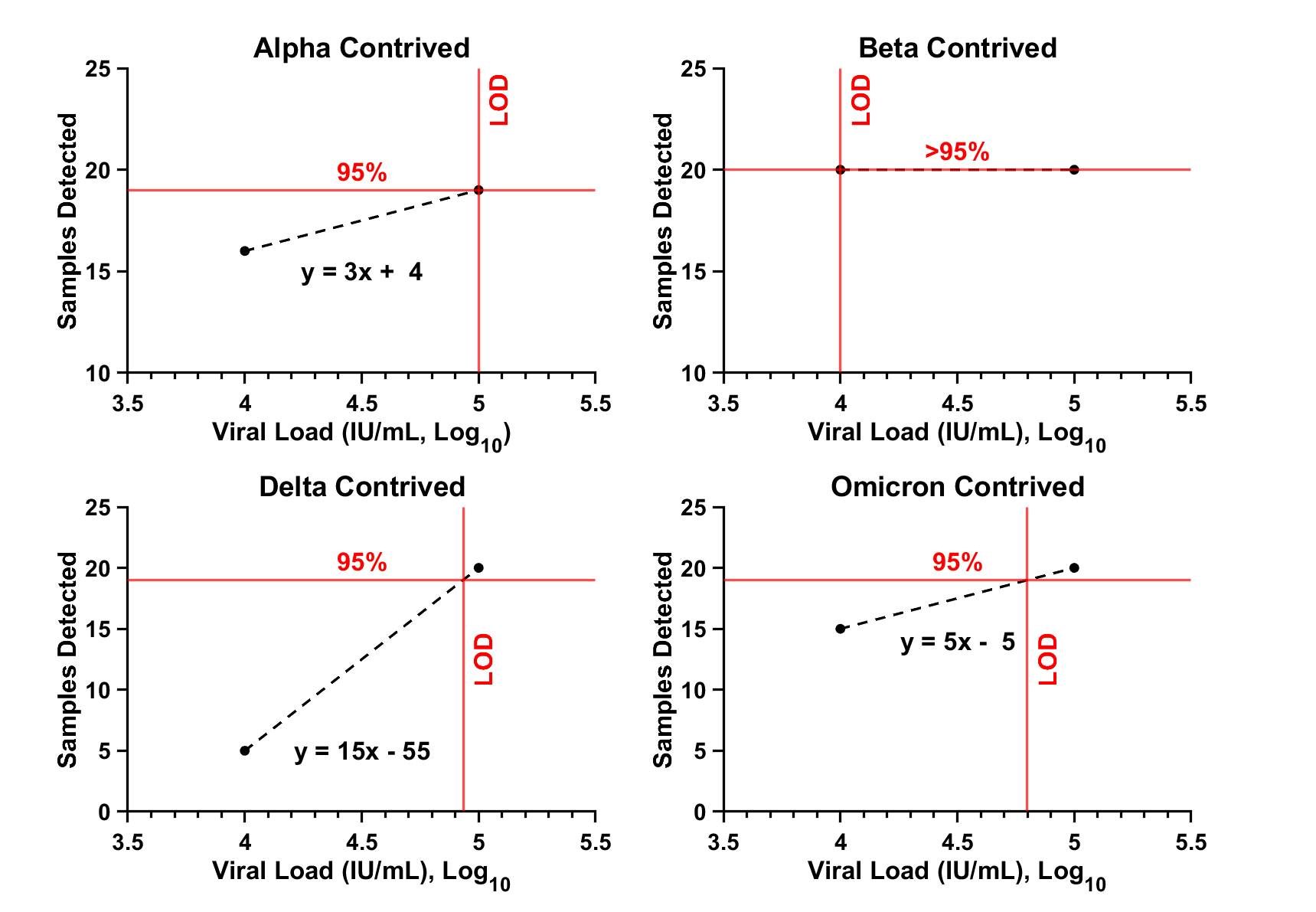


**Figure S3.** Estimations of the variant-typing assay limits of detection for each variant of concern. Approximations were based on a logarithmic-linear regression from experimental data presented in **Table 2**. The limit of detection was determined as the viral load for which 95% or more samples are detectable. For each variant, contrived samples were generated by spiking known-variant inactivated virus into pooled negative nasopharyngeal matrix. Extracts from contrived samples were tested by the variant-typing assay to determine the analytical sensitivity. Two concentrations were evaluated for each variant surrounding the limit of detection and each concentration was evaluated with n = 20 independent samples.

# **Variant-Specific Detection Estimates**


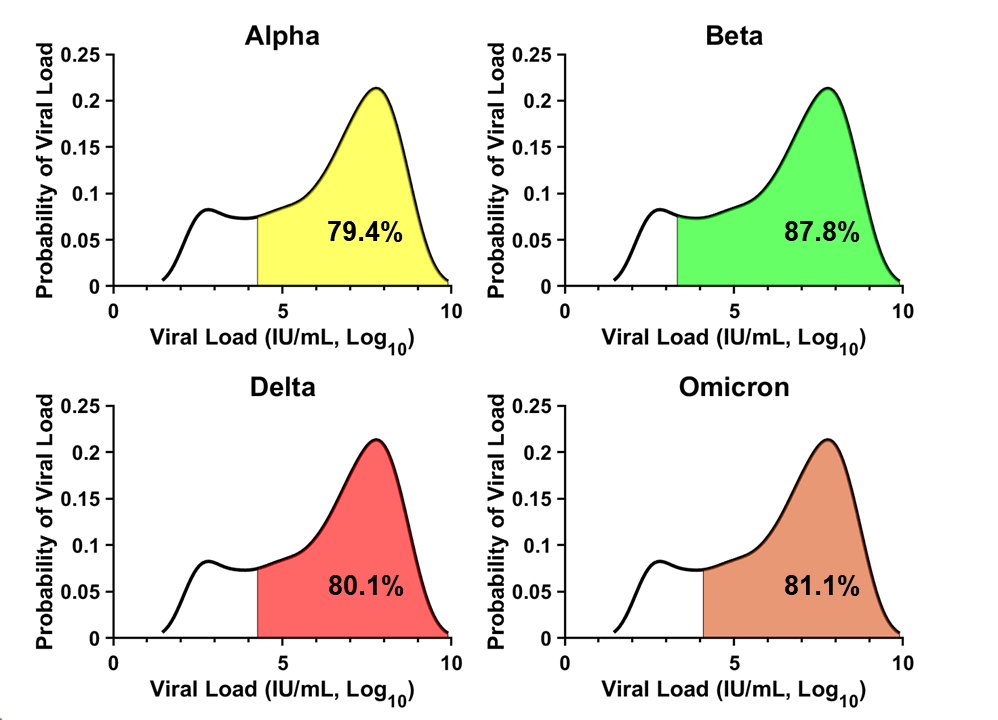


**Figure S4.** Estimations of the variant-typing assay detection performance for untyped, population-level positive COVID-19 clinical samples for each variant-specific platform. Approximations were based on the limit of detection determined by a logarithmic-linear regression from experimental data presented in **Table 2**. The percentage of samples detectable per variant-specific ligation platform (shaded) was determined by comparison to the probability distribution function of the untyped, historically collected clinical samples (black line, n = 5160).

# **Symptomatic Case Variant-Assay Estimate**


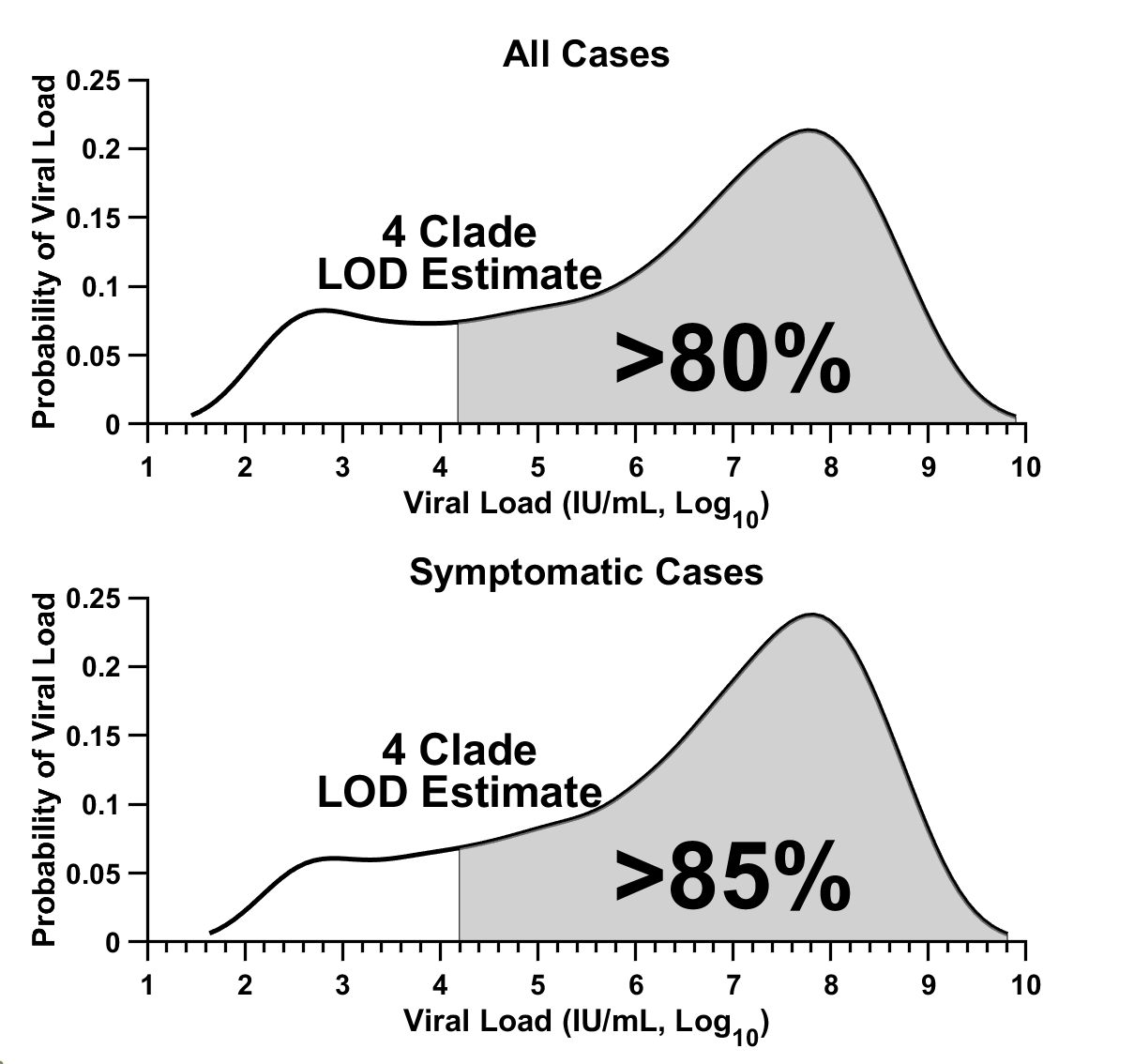


**Figure S5.** Estimations of the variant-typing assay detection performance for untyped, population-level positive COVID-19 clinical samples for all COVID-19 positive cases and symptomatic COVID-19 cases. The variant-typing assay is estimated to detect ~85% of all symptomatic cases (bottom) for all four clades investigated in this work. For all COVID-19 samples, including asymptomatic and test for cure patients, the assay is anticipated to detect approximately 80% of samples.

# **Clinical Sample Viral Load Calculations**

The viral load of clinical samples evaluated in the variant-typing assay studies were determined by RT-PCR performed on a Hologic Panther platform. Each RT-PCR run on the Panther platform included five calibrators of known viral load as internal standards for quantification. The quantification cycle (Cq) values and viral loads for each independent run as well as the linear approximation calculations are provided in the provided Excel (.xlsx) file ‘Panther Calculated Viral Loads’ in the online repository open to the public following publication.

# **Variant-Assay Design Flow Chart**

**
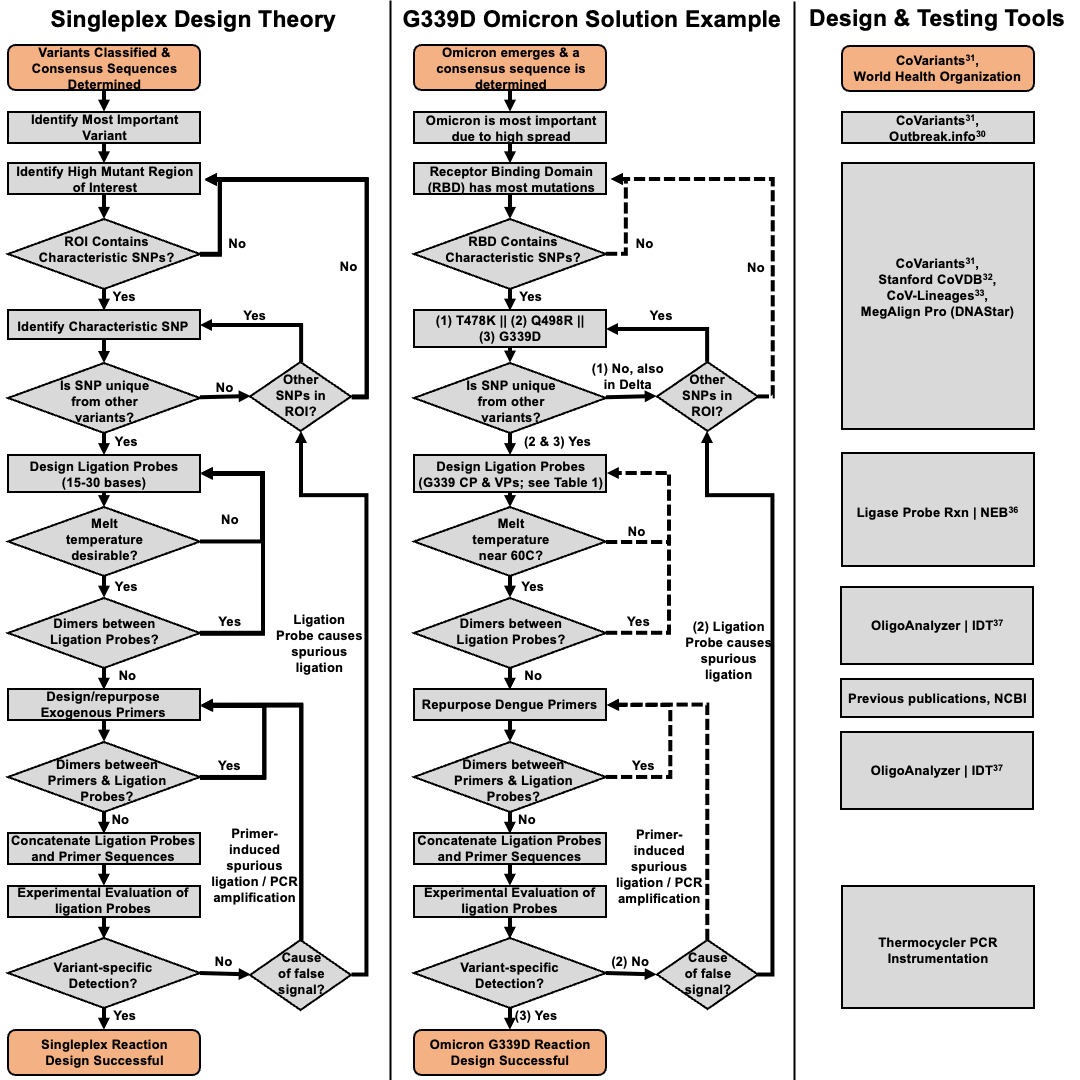
**

**Figure S6.** Flow chart depicting the design process for the oligonucleotide ligation assay that serves as the crux of the variant-typing assay. The design theory is generalizable for other diseases marked by characteristic single nucleotide polymorphisms by conducting a series of design and decision steps (left). In practice, not all pathways are followed, and some iteration will likely be necessary as shown in the example for the Omicron variant-specific detection of the G339D mutation (center). Unfollowed pathways are marked by dotted lines to match the theory. For each step along the design and decision workflow, a combination of different tools and platforms were employed for this specific application to SARS-CoV-2 variants (right). These tools can be used for extension to future SARS-CoV-2 variants, and the general tools can be used to apply this strategy to other diseases. The tools are horizontally aligned with the steps in which they are relevant.

# **Singleplex Clinical Results**

| **Sample** | **Yellow Channel (E484 / Alpha / Pos. Cont)** | **Green Channel (E484K / Beta / Pos. Cont)** | **Red Channel (L452 / Delta)** | **Orange Channel (G339D / Omicron)** | **Assay Classification** | **Actual Classification** | **GISAID** | **Cq Value** | **Viral Load (IU / mL, Log10)** |
| --- | --- | --- | --- | --- | --- | --- | --- | --- | --- |
| **Blind Trial #1 - 5 April 2022** | | | | | | | | | |
| **2928** | **+** | **-** | **+** | **-** | **Delta** | **Delta** | **hCoV-19/USA/TN-VUMC-002928/2021** | 22.6 | 5.87 |
| **2930** | **+** | **-** | **+** | **-** | **Delta** | **Delta** | **hCoV-19/USA/TN-VUMC-002930/2021** | 16.2 | 7.79 |
| **2942** | **+** | **-** | **+** | **-** | **Delta** | **Delta** | **hCoV-19/USA/TN-VUMC-002942/2021** | 16.3 | 7.76 |
| **839** | **+** | **-** | **-** | **-** | **Alpha** | **Alpha** | **hCoV-19/USA/TN-VUMC-000839/2021** | **N/A** |  |
| **840** | **+** | **-** | **-** | **-** | **Alpha** | **Alpha** | **hCoV-19/USA/TN-VUMC-000840/2021** | **N/A** |  |
| **854** | **+** | **-** | **-** | **-** | **Alpha** | **Alpha** | **hCoV-19/USA/TN-VUMC-000854/2021** | **N/A** |  |
| **Blind Trial #2 - 10 June 2022** | | | | | | | | | |
| **DN1** | **+** | **-** | **+** | **+** | **Omicron** | **Omicron** | **EPI_ISL_15856706** | 19.6 | 6.86 |
| **DN2** | **+** | **-** | **+** | **+** | **Omicron** | **Omicron** | **EPI_ISL_15856707** | 19.7 | 6.83 |
| **DN3** | **+** | **-** | **-** | **+** | **Omicron** | **Omicron** | **In Processing for GISAID Submission** | 21.9 | 6.17 |
| **DN4** | **+** | **-** | **+** | **+** | **Omicron** | **Omicron** | **EPI_ISL_15856708** | 16.8 | 7.70 |
| **DN5** | **+** | **-** | **+** | **+** | **Omicron** | **Omicron** | **EPI_ISL_15856709** | 17.3 | 7.55 |
| **DN6** | **+** | **-** | **+** | **+** | **Omicron** | **Omicron** | **EPI_ISL_15856710** | 19 | 7.04 |
| **DN7** | **+** | **-** | **-** | **+** | **Omicron** | **Omicron** | **EPI_ISL_15856711** | 24.8 | 5.30 |
| **DN8** | **+** | **-** | **+** | **+** | **Omicron** | **Omicron** | **EPI_ISL_15856712** | 21.3 | 6.35 |
| **842** | **+** | **-** | **-** | **-** | **Alpha** | **Alpha** | **hCoV-19/USA/TN-VUMC-000842/2021** | **N/A** |  |
| **2937** | **+** | **-** | **+** | **-** | **Delta** | **Delta** | **hCoV-19/USA/TN-VUMC-002937/2021** | 21 | 6.35 |
| **2927** | **+** | **-** | **+** | **-** | **Delta** | **Delta** | **hCoV-19/USA/TN-VUMC-002927/2021** | 21.1 | 6.32 |
| **Blind Trial #3 - 24 Jun 2022** | | | | | | | | | |
| **DN9** | **+** | **-** | **-** | **-** | **Alpha** | **Alpha** | **hCoV-19/USA/TN-VUMC-000715/2021** | 21.2 | 6.35 |
| **DN10** | **+** | **-** | **-** | **-** | **Alpha** | **Alpha** | **hCoV-19/USA/TN-VUMC-000723/2021** | 31.1 | 3.32 |
| **DN11** | **+** | **-** | **-** | **-** | **Alpha** | **Alpha** | **hCoV-19/USA/TN-VUMC-000717/2021** | 19.8 | 6.78 |
| **DN12** | **+** | **-** | **-** | **-** | **Alpha** | **Alpha** | **hCoV-19/USA/TN-VUMC-000725/2021** | 18.9 | 7.06 |
| **DN13** | **+** | **-** | **-** | **-** | **Alpha** | **Alpha** | **hCoV-19/USA/TN-VUMC-000704/2021** | 28.1 | 4.24 |
| **DN14** | **+** | **-** | **+** | **-** | **Delta** | **Delta** | **hCoV-19/USA/TN-VUMC-003001/2021** | 24.8 | 4.80 |
| **DN15** | **+** | **-** | **+** | **-** | **Delta** | **Delta** | **hCoV-19/USA/TN-VUMC-003002/2021** | 24.8 | 4.80 |
| **DN16** | **+** | **-** | **+** | **-** | **Delta** | **Delta** | **hCoV-19/USA/TN-VUMC-003005/2021** | 18.8 | 6.59 |
| **DN17** | **+** | **-** | **+** | **-** | **Delta** | **Delta** | **hCoV-19/USA/TN-VUMC-003006/2021** | 17.2 | 7.06 |
| **DN18** | **+** | **-** | **+** | **-** | **Delta** | **Delta** | **hCoV-19/USA/TN-VUMC-003007/2021** | 20.9 | 5.96 |
| **DN19** | **+** | **-** | **+** | **+** | **Omicron** | **Omicron** | **EPI_ISL_15856713** | 23.3 | 5.75 |
| **DN20** | **+** | **-** | **-** | **+** | **Omicron** | **Omicron** | **EPI_ISL_15856714** | 28.2 | 4.29 |
| **DN21** | **+** | **-** | **+** | **+** | **Omicron** | **Omicron** | **EPI_ISL_15856715** | 19.9 | 6.77 |
| **DN22** | **+** | **-** | **-** | **+** | **Omicron** | **Omicron** | **EPI_ISL_15856716** | 19.4 | 6.92 |
| **DN23** | **+** | **-** | **+** | **+** | **Omicron** | **Omicron** | **EPI_ISL_15856717** | 15.9 | 7.97 |
| **Blind Trial #4 - 28-29 Jun 2022** | | | | | | | | | |
| **DN24** | **+** | **-** | **-** | **-** | **Alpha** | **Alpha** | **hCoV-19/USA/TN-VUMC-000708/2021** | 23.5 | 5.67 |
| **DN25** | **+** | **-** | **-** | **-** | **Alpha** | **Alpha** | **hCoV-19/USA/TN-VUMC-000714/2021** | 17.7 | 7.43 |
| **DN26** | **+** | **-** | **-** | **-** | **Alpha** | **Alpha** | **hCoV-19/USA/TN-VUMC-000720/2021** | 17.9 | 7.37 |
| **DN27** | **+** | **-** | **-** | **-** | **Alpha** | **Alpha** | **hCoV-19/USA/TN-VUMC-000712/2021** | 25.2 | 5.16 |
| **DN28** | **+** | **-** | **-** | **-** | **Alpha** | **Alpha** | **hCoV-19/USA/TN-VUMC-000705/2021** | 30.1 | 3.67 |
| **DN29** | **+** | **-** | **+** | **-** | **Delta** | **Delta** | **hCoV-19/USA/TN-VUMC-003008/2021** | 17.4 | 7.00 |
| **DN30** | **+** | **-** | **+** | **-** | **Delta** | **Delta** | **hCoV-19/USA/TN-VUMC-003009/2021** | 16.8 | 7.18 |
| **DN39** | **+** | **-** | **-** | **-** | **Alpha** | **Alpha** | **hCoV-19/USA/TN-VUMC-000622/2021** | **(-) on Hologic** |  |
| **Blind Trial #5 - 5 Jul 2022** | | | | | | | | | |
| **DN31** | **+** | **-** | **+** | **-** | **Delta** | **Delta** | **hCoV-19/USA/TN-VUMC-003010/2021** | 19.9 | 6.26 |
| **DN32** | **+** | **-** | **+** | **-** | **Delta** | **Delta** | **hCoV-19/USA/TN-VUMC-003011/2021** | 19.3 | 6.44 |
| **DN33** | **+** | **-** | **-** | **-** | **Alpha** | **Delta** | **hCoV-19/USA/TN-VUMC-003015/2021** | 34.5 | 5.16 |
| **DN34** | **+** | **-** | **-** | **+** | **Omicron** | **Omicron** | **EPI_ISL_15856718** | 14.7 | 8.33 |
| **DN35** | **+** | **-** | **+** | **+** | **Omicron** | **Omicron** | **EPI_ISL_15856719** | 23.2 | 5.78 |
| **DN36** | **+** | **-** | **-** | **+** | **Omicron** | **Omicron** | **EPI_ISL_15856720** | 28.3 | 4.26 |
| **DN37** | **+** | **-** | **+** | **+** | **Omicron** | **Omicron** | **EPI_ISL_15856721** | 27.1 | 4.61 |
| **DN38** | **+** | **-** | **+** | **+** | **Omicron** | **Omicron** | **EPI_ISL_15856722** | 24 | 5.54 |
| **Blind Trial #6 - 7 Jul 2022** | | | | | | | | | |
| **DN40** | **+** | **-** | **+** | **-** | **Delta** | **Delta** | **hCoV-19/USA/TN-VUMC-002375/2021** | **N/A** |  |
| **DN41** | **+** | **-** | **-** | **-** | **Alpha** | **Delta** | **hCoV-19/USA/TN-VUMC-002350/2021** | **(-) on Hologic** |  |
| **DN42** | **+** | **-** | **+** | **+** | **Omicron** | **Omicron** | **EPI_ISL_15856732** | 16.6 | 7.78 |
| **DN43** | **+** | **-** | **+** | **-** | **Delta** | **Delta** | **hCoV-19/USA/TN-VUMC-002364/2021** | 17.6 | 7.43 |
| **DN44** | **+** | **-** | **+** | **-** | **Delta** | **Delta** | **hCoV-19/USA/TN-VUMC-002370/2021** | **N/A** |  |
| **DN45** | **+** | **-** | **+** | **-** | **Delta** | **Delta** | **hCoV-19/USA/TN-VUMC-002366/2021** | **N/A** |  |
| **DN46** | **+** | **-** | **+** | **-** | **Delta** | **Delta** | **hCoV-19/USA/TN-VUMC-002356/2021** | 16.5 | 7.76 |
| **DN47** | **+** | **-** | **-** | **-** | **Alpha** | **Alpha** | **hCoV-19/USA/TN-VUMC-000449/2021** | 15.8 | 8.01 |
| **Blind Trial #7 - 11 Jul 2022** | | | | | | | | | |
| **DN48** | **+** | **-** | **-** | **+** | **Omicron** | **Omicron** | **EPI_ISL_15856729** | 20.4 | 6.66 |
| **DN49** | **+** | **-** | **+** | **+** | **Omicron** | **Omicron** | **EPI_ISL_15856730** | 17.5 | 7.51 |
| **DN50** | **+** | **-** | **+** | **+** | **Omicron** | **Omicron** | **EPI_ISL_15856734** | 24.3 | 5.51 |
| **DN51** | **+** | **-** | **-** | **-** | **Alpha** | **Alpha** | **hCoV-19/USA/TN-VUMC-000518/2021** | 19.9 | 6.80 |
| **DN52** | **+** | **-** | **-** | **-** | **Alpha** | **Alpha** | **hCoV-19/USA/TN-VUMC-000528/2021** | 18 | 7.36 |
| **DN53** | **+** | **-** | **-** | **-** | **Alpha** | **Alpha** | **hCoV-19/USA/TN-VUMC-000445/2021** | 17.2 | 7.60 |
| **DN54** | **+** | **-** | **+** | **+** | **Omicron** | **Omicron** | **EPI_ISL_15856731** | 23.2 | 5.83 |
| **DN55** | **+** | **-** | **-** | **-** | **Alpha** | **Alpha** | **hCoV-19/USA/TN-VUMC-000513/2021** | 28.3 | 4.33 |
| **Blind Trial #8 - 20 Jul 2022** | | | | | | | | | |
| **DN56** | **+** | **-** | **-** | **-** | **Alpha** | **Alpha** | **hCoV-19/USA/TN-VUMC-000426/2021** | 25 | 5.24 |
| **DN57** | **+** | **-** | **-** | **-** | **Alpha** | **Alpha** | **hCoV-19/USA/TN-VUMC-000457/2021** | 35.4 | 2.13 |
| **DN58** | **+** | **-** | **-** | **-** | **Alpha** | **Alpha** | **hCoV-19/USA/TN-VUMC-000441/2021** | 34.6 | 2.37 |
| **DN59** | **+** | **-** | **-** | **-** | **Alpha** | **Alpha** | **hCoV-19/USA/TN-VUMC-000526/2021** | 22 | 6.14 |
| **DN60** | **+** | **-** | **+** | **-** | **Delta** | **Delta** | **hCoV-19/USA/TN-VUMC-002361/2021** | 16 | 7.91 |
| **DN61** | **+** | **-** | **+** | **-** | **Delta** | **Delta** | **hCoV-19/USA/TN-VUMC-002358/2021** | 17 | 7.61 |
| **DN62** | **+** | **-** | **+** | **-** | **Delta** | **Delta** | **hCoV-19/USA/TN-VUMC-002137/2021** | 24 | 6.31 |
| **~~DN63~~** | **~~+~~** | **~~-~~** | **~~-~~** | **~~-~~** | **~~Alpha~~** | **REDACTED DUE TO PROCESSING ERROR** | | |  |
| **Blind Trial #9 - 21 Jul 2022** | | | | | | | | | |
| **DN64** | **+** | **-** | **+** | **+** | **Omicron** | **Omicron** | **EPI_ISL_15856741** | 28.2 | 4.29 |
| **DN65** | **+** | **-** | **+** | **-** | **Delta** | **Delta** | **hCoV-19/USA/TN-VUMC-002127/2021** | 22 | 6.14 |
| **DN66** | **+** | **-** | **-** | **-** | **Alpha** | **Alpha** | **hCoV-19/USA/TN-VUMC-000408/2021** | 28.1 | 4.32 |
| **DN67** | **+** | **-** | **+** | **+** | **Omicron** | **Omicron** | **EPI_ISL_15856740** | 26 | 4.94 |
| **DN68** | **+** | **-** | **+** | **+** | **Omicron** | **Omicron** | **EPI_ISL_15856739** | 16.1 | 7.91 |
| **DN69** | **+** | **-** | **-** | **-** | **Alpha** | **Alpha** | **hCoV-19/USA/TN-VUMC-000522/2021** | 18.4 | 7.22 |
| **DN70** | **+** | **-** | **-** | **-** | **Alpha** | **Delta** | **hCoV-19/USA/TN-VUMC-002183/2021** | 27.6 | 4.47 |
| **DN71** | **+** | **-** | **+** | **+** | **Omicron** | **Omicron** | **In Processing for GISAID Submission** | 16.8 | 7.70 |
| **Blind Trial #10 - 22 Jul 2022** | | | | | | | | | |
| **DN72** | **+** | **-** | **-** | **-** | **Alpha** | **Delta** | **hCoV-19/USA/TN-VUMC-002179/2021** | **(-) on Hologic** |  |
| **DN73** | **+** | **-** | **+** | **-** | **Delta** | **Delta** | **hCoV-19/USA/TN-VUMC-002154/2021** | 20 | 6.68 |
| **DN74** | **+** | **-** | **+** | **-** | **Delta** | **Delta** | **hCoV-19/USA/TN-VUMC-002123/2021** | 24.4 | 5.42 |
| **DN75** | **+** | **-** | **-** | **-** | **Alpha** | **Alpha** | **hCoV-19/USA/TN-VUMC-000500/2021** | 30 | 3.75 |
| **DN76** | **-** | **-** | **-** | **-** | **No Signal** | **Omicron** | **EPI_ISL_15856737** | **(-) on Hologic** |  |
| **DN77** | **+** | **-** | **-** | **-** | **Alpha** | **Alpha** | **hCoV-19/USA/TN-VUMC-000405/2021** | 18.5 | 7.19 |
| **DN78** | **+** | **-** | **+** | **+** | **Omicron** | **Omicron** | **EPI_ISL_15856736** | 18.2 | 7.28 |
| **DN79** | **+** | **-** | **-** | **-** | **Alpha** | **Alpha** | **hCoV-19/USA/TN-VUMC-000401/2021** | 19.9 | 6.77 |
| **DN80** | **+** | **-** | **+** | **+** | **Omicron** | **Omicron** | **EPI_ISL_15856735** | 20.1 | 6.71 |
| **DN81** | **+** | **-** | **-** | **-** | **Alpha** | **Alpha** | **hCoV-19/USA/TN-VUMC-000505/2021** | 31.7 | 3.24 |
| **DN82** | **+** | **-** | **+** | **-** | **Delta** | **Delta** | **hCoV-19/USA/TN-VUMC-002198/2021** | 19.1 | 7.01 |
| **DN83** | **+** | **-** | **-** | **-** | **Alpha** | **Delta** | **hCoV-19/USA/TN-VUMC-002168/2021** | **(-) on Hologic** |  |
| **DN84** | **+** | **-** | **+** | **-** | **Delta** | **Delta** | **hCoV-19/USA/TN-VUMC-002197/2021** | 22.8 | 5.90 |
| **DN85** | **+** | **-** | **-** | **+** | **Omicron** | **Delta** | **hCoV-19/USA/TN-VUMC-002402/2021** | 20 | 6.74 |

**Table S4. Summary of clinical trials with the singleplex variant-typing assay.**

# **Multiplex Clinical Results**

| **Sample** | **NGS Classification** | **Multiplexed Classification** | **Multiplexed Experiment / Date** | **Sample** | **NGS Classification** | **Multiplexed Classification** | **Multiplexed Experiment / Date** |
| --- | --- | --- | --- | --- | --- | --- | --- |
| **2928** | **Delta** | **Delta** | 8/23/2022 | **DN39** | **(-) on Hologic** |  |  |
| **2930** | **Delta** | **Delta** | 8/23/2022 | **DN40** | **Delta** | **Delta** | 8/22/2022 |
| **2942** | **Delta** | **Delta** | 8/23/2022 | **DN41** | **(-) on Hologic** |  |  |
| **839** | **Alpha** | **Alpha** | 8/23/2022 | **DN42** | **Omicron** | **Omicron** | 8/22/2022 |
| **840** | **Alpha** | **Alpha** | 8/23/2022 | **DN43** | **Delta** | **Delta** | 8/22/2022 |
| **854** | **Alpha** | **Alpha** | 8/23/2022 | **DN44** | **Delta** | **Delta** | 8/22/2022 |
| **DN1** | **Omicron** | **Omicron** | 8/23/2022 | **DN45** | **Delta** | **Delta** | 8/22/2022 |
| **DN2** | **Omicron** | **Omicron** | 8/23/2022 | **DN46** | **Delta** | **Delta** | 8/22/2022 |
| **DN3** | **Omicron** | **Omicron** | 8/23/2022 | **DN47** | **Alpha** | **Alpha** | 8/22/2022 |
| **DN4** | **Omicron** | **Omicron** | 8/23/2022 | **DN48** | **Omicron** | **Omicron** | 8/22/2022 |
| **DN5** | **Omicron** | **Omicron** | 8/23/2022 | **DN49** | **Omicron** | **Omicron** | 8/22/2022 |
| **DN6** | **Omicron** | **Omicron** | 8/23/2022 | **DN50** | **Omicron** | **Omicron** | 8/22/2022 |
| **DN7** | **Omicron** | **Omicron** | 8/23/2022 | **DN51** | **Alpha** | **Alpha** | 8/22/2022 |
| **DN8** | **Omicron** | **Omicron** | 8/23/2022 | **DN52** | **Alpha** | **Alpha** | 8/9/2022 |
| **842** | **Alpha** | **Alpha** | 8/23/2022 | **DN53** | **Alpha** | **Alpha** | 8/9/2022 |
| **2937** | **Delta** | **Delta** | 8/23/2022 | **DN54** | **Omicron** | **Omicron** | 8/9/2022 |
| **2927** | **Delta** | **Delta** | 8/23/2022 | **DN55** | **Alpha** | **Alpha** | 8/9/2022 |
| **DN9** | **Alpha** | **Alpha** | 8/23/2022 | **DN56** | **Alpha** | **Alpha** | 8/9/2022 |
| **DN10** | **Alpha** | **Alpha** | 8/22/2022 | **DN57** | **Alpha** | **No Signal** | 8/9/2022 |
| **DN11** | **Alpha** | **Alpha** | 8/22/2022 | **DN58** | **Alpha** | **No Signal** | 8/9/2022 |
| **DN12** | **Alpha** | **Alpha** | 8/22/2022 | **DN59** | **Alpha** | **Alpha** | 8/9/2022 |
| **DN13** | **Alpha** | **Alpha** | 8/22/2022 | **DN60** | **Delta** | **Delta** | 8/9/2022 |
| **DN14** | **Delta** | **Delta** | 8/22/2022 | **DN61** | **Delta** | **Delta** | 8/9/2022 |
| **DN15** | **Delta** | **Delta** | 8/22/2022 | **DN62** | **Delta** | **Delta** | 8/9/2022 |
| **DN16** | **Delta** | **Delta** | 8/22/2022 | **~~DN63~~** | **Processing Error** |  |  |
| **DN17** | **Delta** | **Delta** | 8/22/2022 | **DN64** | **Omicron** | **Delta** | 8/8/2022 |
| **DN18** | **Delta** | **Delta** | 8/22/2022 | **DN65** | **Delta** | **Delta** | 8/8/2022 |
| **DN19** | **Omicron** | **Omicron** | 8/22/2022 | **DN66** | **Alpha** | **Alpha** | 8/8/2022 |
| **DN20** | **Omicron** | **Omicron** | 8/22/2022 | **DN67** | **Omicron** | **Omicron** | 8/8/2022 |
| **DN21** | **Omicron** | **Omicron** | 8/22/2022 | **DN68** | **Omicron** | **Omicron** | 8/8/2022 |
| **DN22** | **Omicron** | **Omicron** | 8/22/2022 | **DN69** | **Alpha** | **Alpha** | 8/8/2022 |
| **DN23** | **Omicron** | **Omicron** | 8/22/2022 | **DN70** | **Delta** | **Delta** | 8/3/2022 |
| **DN24** | **Alpha** | **Alpha** | 8/22/2022 | **DN71** | **Omicron** | **Omicron** | 8/8/2022 |
| **DN25** | **Alpha** | **Alpha** | 8/22/2022 | **DN72** | **(-) on Hologic** |  |  |
| **DN26** | **Alpha** | **Alpha** | 8/22/2022 | **DN73** | **Delta** | **Delta** | 8/8/2022 |
| **DN27** | **Alpha** | **Alpha** | 8/22/2022 | **DN74** | **Delta** | **Delta** | 8/8/2022 |
| **DN28** | **Alpha** | **Alpha** | 8/22/2022 | **DN75** | **Alpha** | **No Signal** | 8/8/2022 |
| **DN29** | **Delta** | **Delta** | 8/22/2022 | **DN76** | **(-) on Hologic** |  |  |
| **DN30** | **Delta** | **Delta** | 8/22/2022 | **DN77** | **Alpha** | **Alpha** | 8/8/2022 |
| **DN31** | **Delta** | **Delta** | 8/22/2022 | **DN78** | **Omicron** | **Omicron** | 8/3/2022 |
| **DN32** | **Delta** | **Delta** | 8/22/2022 | **DN79** | **Alpha** | **Alpha** | 8/2/2022 |
| **DN33** | **Delta** | **Delta** | 8/22/2022 | **DN80** | **Omicron** | **Omicron** | 8/8/2022 |
| **DN34** | **Omicron** | **Omicron** | 8/22/2022 | **DN81** | **Alpha** | **No Signal** | 8/8/2022 |
| **DN35** | **Omicron** | **Omicron** | 8/22/2022 | **DN82** | **Delta** | **Delta** | 8/2/2022 |
| **DN36** | **Omicron** | **Omicron** | 8/22/2022 | **DN83** | **(-) on Hologic** |  |  |
| **DN37** | **Omicron** | **Omicron** | 8/22/2022 | **DN84** | **Delta** | **Delta** | 8/8/2022 |
| **DN38** | **Omicron** | **Omicron** | 8/22/2022 | **DN85** | **Delta** | **Omicron** | 8/3/2022 |

**Table S5. Summary of clinical trials with the multiplex variant-typing assay.** Multiplex trials were performed following the singleplex trials, therefore, known negative samples were not peformed on the multiplex platform as indicated by black boxes.
